# Cell Rover—a miniaturized magnetostrictive antenna for wireless operation inside living cells

**DOI:** 10.1038/s41467-022-32862-4

**Published:** 2022-09-22

**Authors:** Baju Joy, Yubin Cai, David C. Bono, Deblina Sarkar

**Affiliations:** 1 MIT Media Lab, Massachusetts Institute of Technology, Cambridge, MA 02139 USA; 2 Department of Materials Science and Engineering, Massachusetts Institute of Technology, Cambridge, MA 02139 USA

**Keywords:** Electrical and electronic engineering, Biomedical engineering

## Abstract

An intracellular antenna can open up new horizons for fundamental and applied biology. Here, we introduce the Cell Rover, a magnetostrictive antenna which can operate wirelessly inside a living cell and is compatible with 3D biological systems. It is sub-mm in size, acoustically actuated by an AC magnetic field and resonantly operated at low MHz frequencies, which is ideal for living systems. We developed an injection scheme involving non-uniform magnetic fields for intracellular injection of the Cell Rovers and demonstrated their operation in fully opaque, stage VI Xenopus oocytes, for which real-time imaging with conventional technologies is challenging. We also show that they provide a pathway for multiplexing applications to individually address multiple cells or to tune to more than one antenna within the same cell for versatile functionalities. This technology forms the foundation stone that can enable the integration of future capabilities such as smart sensing, modulation as well as energy harvesting to power in-cell nanoelectronic computing and can potentially bring the prowess of information technology inside a living cell. This could lead to unprecedented opportunities for fundamental understanding of biology as well as diagnostics and therapeutics.

## Abstract (web summary)

An intracellular antenna that can truly enable probing, modulation or augmentation of biological cells remains an unmet challenge. Here, the authors present the Cell Rover, a magnetostrictive antenna that operates wirelessly inside a living cell and is compatible with 3D biological systems.

## Introduction

Cells are complex living machines and form the building blocks of our biological system. Probing and manipulation at the cellular level can provide fundamental insights into biology and disease as well as lead to advanced health monitoring and treatment options. To achieve this, a variety of techniques have been developed, involving introduction of different extraneous agents into the cells. These agents can be of biological origin such as proteins^1, 2^, RNA^3, 4^, DNA^5, 6^ or inorganic such as engineered devices/particles^7, 8^ and fluorescent dyes^9, 10^. An antenna which can operate inside a living cell is a promising alternative to the existing techniques and can enable functionalities not achievable with other technologies. It can not only be used for wireless intracellular sensing, modulation and cell tracking but also for energy harvesting to power active nanoelectronic devices, which enabled by 50 years of advancement in information technology, provide an extreme combination of complexity, accuracy and speed. These nanoelectronic devices can be employed for in-situ computing and information processing to achieve smart exploration and manipulation of intracellular environment as well as inter-cellular communication, leading to unprecedented opportunities. In addition, an antenna is not limited to optically accessible systems only and can work well in opaque cells, 3D biological systems and in-vivo. However, miniaturization of an antenna to fit inside a living cell has significant challenges. Conventional electromagnetic (EM) antennas work on the principle of electromagnetic resonance and need to have sizes comparable to the wavelength of the radiated EM wave^11^. This means that far field detection of sub-mm antennas requires operation at 100 s of GHz or THz frequencies which precludes extension of their application in vivo, due to high signal loss and heating effects^12^. Also, electrically small sub-mm EM antennas operating using near field inductive coupling have a short detection range and operate at GHz frequencies which leads to adverse heating effects in living systems^12^. Hence, an intracellular antenna has been rarely demonstrated so far; the only previous report^13^ has a short detection range of 25 μm and an *R/D* value of 1 (where, $\frac{R}{D}=\frac{{{{{{\rm{Distance}}}}}}\; {{{{{\rm{between}}}}}}\; {{{{{\rm{antenna}}}}}}\; {{{{{\rm{and}}}}}}\; {{{{{\rm{receiver}}}}}}}{{{{{{\rm{Effective}}}}}}\; {{{{{\rm{size}}}}}}\; {{{{{\rm{of}}}}}}\; {{{{{\rm{antenna}}}}}}}$, is the normalized detection range). This limits its operation to 2D cultured cells with the further constraint that the cell has to be physically taped onto the receiver for wireless detection making it unsuitable for applications involving interacting cells or 3D biological systems such as Xenopus oocytes (1.2 mm in diameter), 3D cellular networks, organoids and in-vivo systems. Moreover, it operates at 60 GHz which limits the maximum power that can be safely transmitted to them without causing adverse heating effects^12^. Due to tissue heating, low MHz frequencies have been shown to be optimum for delivering maximum power for in-vivo operation^14^. However, fabricating a sub-mm EM antenna which can be resonantly operated at low MHz frequencies is practically difficult^15^. Hence, an intracellular antenna that can truly enable investigation and modulation of cells, for fundamental or applied biology, remains an unmet challenge till date.

**Fig. 1:**
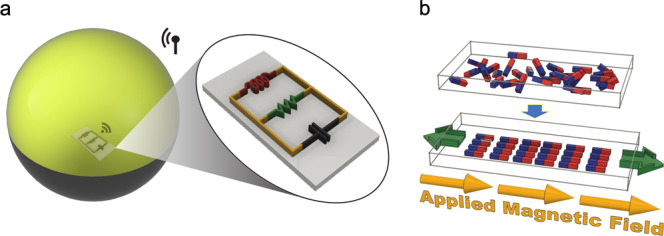
Schematic representation and operating principle of the Cell Rover. **a** Schematic diagram showing the wireless operation of a Cell Rover from inside a cell (Xenopus oocyte). The zoomed in view shows the Cell Rover and its equivalent circuit representation as a parallel RLC resonator. **b** Schematic diagram illustrating the principle of magnetostriction. The red and blue faces indicate north and south poles of the magnetic domains in the material respectively. The randomly oriented magnetic domains align in the direction of an applied magnetic field which in turn causes a strain in the material.

Here we develop the Cell Rover (Fig. 1a), a magnetostrictive antenna that can work inside a living cell, be remotely operated and is compatible with 3D biological systems. It is sub-mm in size (500 μm × 200 μm × 28 μm dimensions and volume < 0.003 ${{mm}}^{3}$), has a detection range of 1.0 cm (*R/D* = 28.0) and has a frequency of operation of few MHz which is optimal for working in living systems. It works on the principle of magnetostriction ($\lambda$) in which a material experiences a strain in response to magnetization and vice versa as illustrated by Fig. 1b. When excited with an AC magnetic field acoustic waves are produced in the material and the resulting magnetization can be wirelessly detected by near-field inductive coupling through a coil. Since for a given frequency the acoustic wavelength in the magnetostrictive material is about five orders of magnitude smaller than the EM wavelength, magnetostrictive antennas can have orders of magnitude smaller sizes compared to conventional EM antennas^16–18^. However, a sub-mm magnetostrictive antenna which works within a living cell or for that matter any living system has not been demonstrated previously.

We demonstrate the operation of the Cell Rover in Stage VI Xenopus Laevis oocytes which are a common model used to study cell biology, developmental biology, and modeling human disease^19–21^. Stage VI Xenopus oocytes have a diameter of about 1.2 mm, are fully opaque and have a high concentration of yolk platelets which show strong autofluorescence. The large spherical size and opaque nature of the oocytes makes it difficult to perform live imaging of whole oocytes^22^. Moreover, due to autofluorescence and attenuation from deeper regions of the cytoplasm live fluorescence imaging is difficult except in the cortical cytoplasm^23^. On the other hand, intracellular electrodes which are used for stimulation and sensing of ions in Xenopus oocytes suffer from the disadvantage that they are wired which precludes any measurements when the cell is not stationary. Furthermore, techniques such as western blotting and histological methods are not real time and involves destruction of the cells. Cell Rovers can overcome these limitations of existing technologies and can be highly advantageous in the investigation and manipulation of these cells and thus, open up new avenues for studying fundamental biology and lead to novel healthcare applications.

Here, first, we fabricate the Cell Rovers and characterize them in both dry and wet environment to study the frequency response, mass loading and viscous damping effects. Then, an injection scheme employing optimized magnetic fields is developed to achieve minimally invasive injection of these antennas into Xenopus oocytes. This is followed by demonstration of their wireless detection from inside the cells and characterization of the frequency response. We further show that Cell Rovers of different sizes can be successfully used in living cells for multiplexing.

## Results

### Fabrication and characterization of Cell Rovers

**Fig. 2:**
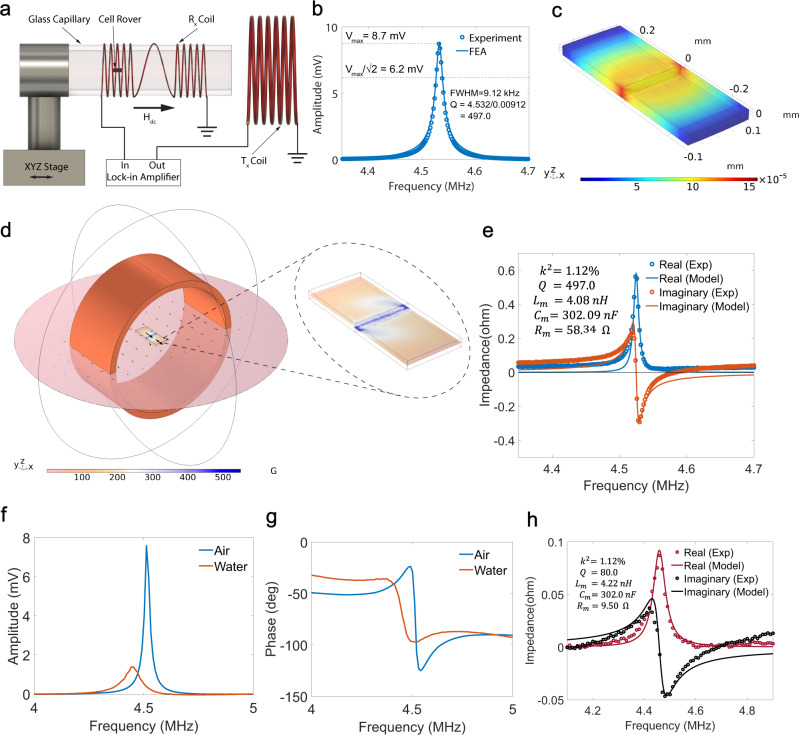
Characterization of Cell Rovers in air and water. **a** Schematic diagram showing the wireless detection of a Cell Rover using a receiving $({R}_{x})$ coil consisting of two identical but oppositely wound solenoids connected to a lock-in amplifier. The transmission $({T}_{x})$ coil generates the AC excitation magnetic field and a permanent magnet is used to produce the required DC bias magnetic field. **b** Comparison between measured and FEA simulated wirelessly detected voltage amplitude from a Cell Rover in air as a function of frequency of excitation magnetic field. The signal amplitude is maximum $({V}_{{\max }})$ at the resonance frequency (4.532 MHz). The calculation for the quality factor (Q) from the Full Width at Half Maximum (FWHM) is also shown. **c** FEA simulation of the distribution of strain in the Cell Rover at the resonance frequency (4.532 MHz). **d** FEA simulation showing the magnetic flux density distribution in the ${R}_{x}$ coil containing the Cell Rover at the resonance frequency (4.532 MHz). A zoomed in view of the mid-plane of the resonator is also shown. **e** Impedance vs Frequency of the Cell Rover in air measured using a Vector Network Analyzer (VNA) and the corresponding equivalent circuit model fit which gives a mechanical quality factor (Q) of 497.0 and a magnetomechanical coupling coefficient (${k}^{2}$) of 1.12%. The calculated values for motional inductance $({L}_{m})$, motional capacitance (${C}_{m}$), and motional resistance (${R}_{m}$) are also shown. Comparison of measured **f** voltage amplitude and **g** phase of the Cell Rover in air and water as a function of frequency of excitation magnetic field. **h** Impedance vs Frequency of the Cell Rover in water measured using a VNA and the corresponding equivalent circuit model fit which gives a resonance frequency of 4.452 MHz, quality factor of 80.0 and magnetomechanical coupling coefficient (${k}^{2}$) of 1.12%. All measurements shown are for a Cell Rover of dimension 500 μm × 200 μm × 28 μm at optimum bias magnetic field of 125 Oe.

We have chosen the amorphous magnetostrictive material Metglas 2826 MB for fabricating the Cell Rovers due to its high reported magnetomechanical coupling coefficient^24^, which denotes the efficiency of conversion of input magnetic energy to mechanical energy (Supplementary Note 1). Cell Rovers of dimensions ranging from 400 μm × 200 μm × 28 μm to 625 μm × 200 μm × 28 μm were fabricated by micromachining of a 28 μm thick film of Metglas 2826 MB using a laser or Diesaw as described in the methods section (See Supplementary Fig. 2 and Supplementary Note 2 for details). The volume of the fabricated antennas is <0.05 % of the total cell volume, which is much below the acceptable limit for cell intrusiveness^25^. Figure 2a shows a schematic representation of the experimental setup for wireless detection of Cell Rovers. It consists of a transmission (Tx) coil to excite the magnetostrictive antenna with a uniform magnetic field and a receiving (Rx) coil for the detection of the induced magnetization. The Rx coil was designed as a first-order gradiometer, consisting of two identical coils, connected in series and wound in opposite directions positioned symmetrically to the Tx coil during measurement using the micromanipulator stage (See methods section for details on coil design). The amplitude and phase of the voltage across the Rx coil as a function of frequency of the excitation magnetic field was measured using a lock-in amplifier. The amplitude of oscillation of the resonator in response to a small AC magnetic field excitation depends on the magnetostrictivity ($d=\frac{d\lambda }{{dH}}$) of the material which is the slope of the magnetostriction ($\lambda$) vs magnetic field strength (H) curve as shown in Supplementary Fig. 1a (Supplementary Note 1). An optimum DC bias magnetic field that maximizes the magnetostrictivity (Supplementary Fig. 1b) and hence the detected voltage is applied to the material using a permanent magnet. Finite Element Analysis (FEA) modeling using Comsol Multiphysics was performed to study the frequency response of Cell Rovers and extract values of important parameters such as, magnetostrictivity ($d$), Young’s modulus ($E$) and Rayleigh damping coefficient ($\alpha$) (which characterizes the structural damping in the mechanical resonator) (See Methods section, Supplementary Note 3, Supplementary Fig. 3 and Supplementary Movie 1 for details).

When a mechanical resonator is operated in cell cytoplasm there is a decrease in the mechanical resonance frequency due to mass loading and decrease in quality factor due to viscous damping effects compared to operation in air. Since water has a viscosity comparable to cell cytoplasm^26^ it is expected that the detected response in water would be a good estimate of the resonator response when it is placed in cell cytoplasm. Hence, the Cell Rovers were first characterized in air and water to study the frequency response and understand the effect of mass loading and viscous damping. A Cell Rover of dimensions 500 μm × 200 μm × 28 μm, excited by an AC magnetic field of 3 Oe applied along its length, was used for all measurements and simulations in this section. The optimum DC bias magnetic field, also applied along the length, at which the magnetostrictivity is highest was found to be 125 Oe for a resonator of this size.

Figure 2b shows the simulated and measured coil voltage as a function of frequency when the Cell Rover is present inside the Rx coil, which are in good agreement. The values of magnetostrictivity ($d$), Young’s modulus ($E$), Rayleigh damping coefficient ($\alpha$) and other material properties obtained are shown in Supplementary Table 1. At the mechanical resonance frequency, the strain and hence magnetization in the resonator is maximum thereby leading to a peak in the detected coil voltage at resonance. The mechanical resonance frequency (${f}_{r}$) is found to be 4.532 MHz (standard deviation = 0.016 MHz, number of samples *n* = 5) for a 500 μm × 200 μm × 28 μm resonator which is the natural frequency of vibration of a thin film resonator of length L excited in the longitudinal mode and is roughly given by the formula:^17^

1 $${f}_{r}=\frac{1}{2L}\sqrt{\frac{E}{\rho (1-\nu )}}$$

where $E$ is the Young’s modulus, $\rho$ is the density and $\nu$ is the Poisson’s ratio of the material. Operation in the low MHz frequency range makes Cell Rovers more suitable for near field inductive coupling at larger distances. Supplementary Fig. 4 shows the measured signal amplitude and noise level as a function of the distance from the Cell Rover to the receiving (Rx) coil. It is observed that the Cell Rovers can be detected at a distance of up to 1.0 cm outside the Rx coil with signal to noise ratio of 19.5 dB (normalized detection range (*R/D*) value of 28.0), making them suitable for wireless detection in 3D biological systems (Supplementary Note 4). Figure 2c shows the simulated strain distribution and Fig. 2d shows the simulated magnetic flux density distribution when the Cell Rover is present inside the Rx coil at the resonance frequency (4.532 MHz).

To characterize the impedance and wireless power transfer efficiency of the Cell Rover device, we consider a Cell Rover which is coupled to a single Tx coil of diameter 2 mm, length 1 mm and 26 turns as shown in Supplementary Fig. 5a. Impedance of the resonator and wireless power transfer efficiency is calculated by measuring the reflection coefficient (S11) of the Tx coil with and without the resonator present inside it using a vector network analyzer (VNA) (See methods section for details on the measurement setup). This approach was used since unlike conventional EM antennas, magnetostrictive antennas convert received power into strain energy due to which the conventional two-port S-parameter analysis is not possible. Supplementary Fig. 5b and 5c shows the measured S11 response of the Tx coil with and without the resonator inside it. The equivalent resonator impedance was then calculated using the equivalent circuit model for a magnetostrictive resonator coupled with a coil^27^ (Supplementary Note 5). The mechanical resonance behavior of the resonator is modeled as a parallel LCR circuit as shown in Supplementary Fig. 6, where ${L}_{m}$, ${C}_{m}$, ${R}_{m}$ denote the motional inductance, motional capacitance and motional resistance which characterizes the inertia, elasticity and mechanical losses in the resonator respectively. The resonator impedance obtained from experiment and that for the equivalent circuit model for the best fit parameters are shown in Fig. 2e. The values obtained for these parameters are also shown in Fig. 2e. ${L}_{m}$ is proportional to the mass whereas ${R}_{m}$ and ${C}_{m}$ are inversely proportional to the damping and stiffness of the resonator respectively. From the model, the resonator is also found to have a magnetomechanical coupling coefficient (${k}^{2}$) of 1.12 %, and a high mechanical Q-factor (*Q*) (median = 497.0, standard deviation = 103.6, number of samples, *n* = 5) for vibration in air. ${k}^{2}$ denotes the efficiency of conversion of input magnetic energy into strain energy and *Q* denotes the ratio of energy stored to the energy dissipated in one cycle of oscillation of the resonator. Using the same S11 data the total coupling coefficient and wireless power transfer efficiency of the Cell Rover in air was calculated by modeling the Cell Rover and Tx coil system as two inductively coupled solenoids (Supplementary Fig. 7a) with total coupling coefficient (${k}_{t}$) as detailed in Supplementary Note 6. A ${k}_{t}$ of 0.0066 and wireless power transfer efficiency of 3.67%, assuming maximum power transfer condition, was obtained for the resonator in air (Supplementary Table 3). Supplementary Fig. 7b shows the measured S11 response of the Tx coil containing the resonator and the S11 response of the model for the best fit parameters. The total coupling coefficient ${k}_{t}$ is much lower than the magnetomechanical coupling coefficient (${k}^{2}$) due to the weak inductive coupling which arises from the large difference in the dimensions of the transmitting coil with respect to the Cell Rover and can be improved significantly with a more optimized coil design for in-vivo applications. The current transmitting coil’s dimensions (2 mm in diameter and 1 mm in length) are chosen to enable measurements in Xenopus oocyte cells, which are about 1.2 mm in diameter.

To compare the resonator response in water with that in air, the frequency response of the fabricated antennas was detected in water using the same setup as in Fig. 2a by filling the capillary tube containing the antenna with water. Figure 2f, g shows the comparison of the measured coil voltage amplitude and phase respectively in water vs those in air as a function of frequency. The impedance of the resonator in water is also measured using a VNA with a single Tx coil as done for measurement in air. Figure 2h shows the measured resonator impedance and calculated impedance from the equivalent circuit fit. The resonance frequency has decreased from 4.532 MHz in air to 4.452 MHz (standard deviation = 0.018 MHz, number of samples *n* = 5) in water due to mass loading effects and the Q-factor has decreased from 497.0 to 80.0 (standard deviation = 4.1, number of samples *n* = 5) due to viscous damping. The mass loading and viscous damping effects are represented by the larger motional inductance (lower resonance frequency) and lower motional resistance (lower Q) in the equivalent circuit model (Supplementary Table 2). The magnetomechanical coupling coefficient is the same in water as that in air since it is a property of the resonator material. The total coupling coefficient (${k}_{t}$) and wireless power transfer efficiency for operation in water was also calculated to be 0.0066 and 0.62% respectively. ${k}_{t}$ remains the same but the wireless power transfer efficiency has reduced due to the decrease in quality factor in water. Supplementary Fig. 7d shows the measured S11 response for the Tx coil containing the resonator in water and the S11 response of the model for the best fit parameters.

The resonator response was also modeled using 1D analytical equations to characterize the observed mass loading and viscous damping effects as shown in Supplementary Note 7. Mass loading effect arises from a thin layer of liquid close to the resonator surface which is activated by its vibration which increases the effective mass of the resonator and this increase is characterized by the parameter ${m}_{w}$. This additional mass load leads to the observed decrease in the resonance frequency in water compared to air. Viscous damping is caused due to significant resistive forces acting on the resonator due to much larger viscosity of water compared to air thereby reducing the quality factor, which is characterized by the damping parameter ${c}_{{visc}}$. Supplementary Fig. 8b shows the measured and simulated response from the analytical model for vibration in air and water and Supplementary Table 4 shows the obtained values for parameters ${m}_{w}$ and ${c}_{{visc}}$ for vibration in water. These effects are assumed to be negligible for vibration in air. The resonator response in water shows that Cell Rovers can still operate in a viscous medium like water although the quality factor is decreased due to viscous damping. To check whether the resonator performance is degraded over time in wet medium due to electrochemical reactions, the Cell Rovers were incubated in Phosphate Buffered Saline (PBS) for a period of 5 days and the resonator response was measured. PBS mimics the cell cytoplasm environment since it has a similar concentration of ions^28^. The variation in detected voltage is negligible, <0.5 mV (about 5.9 % of the maximum measured voltage amplitude) which shows that the Cell Rovers can function in wet medium over long periods of time without degradation (Supplementary Fig. 9).

### Intracellular injection of Cell Rovers into Xenopus oocytes

**Fig. 3:**
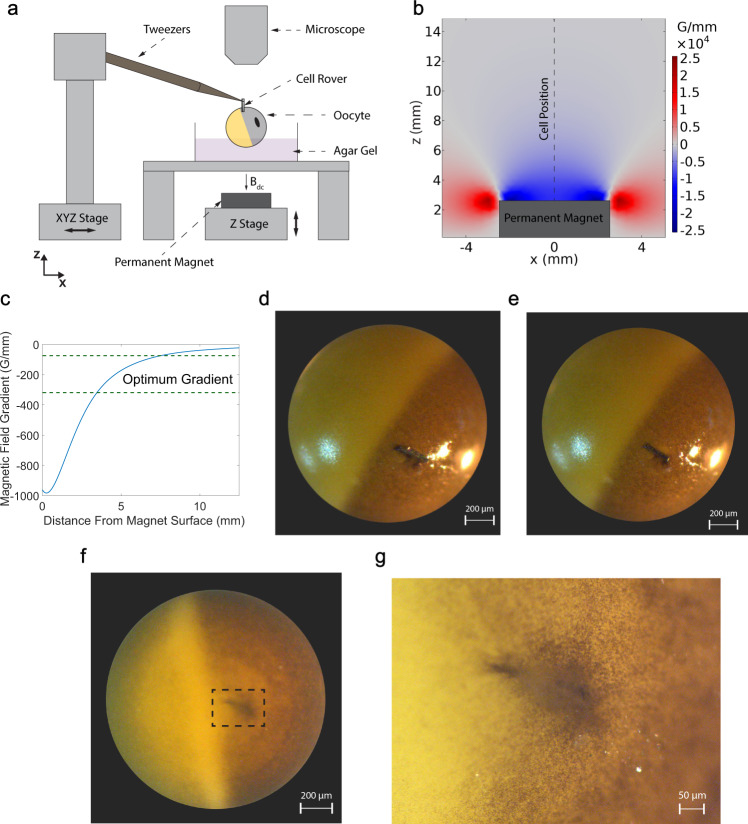
Intracellular injection of Cell Rovers. **a** Schematic diagram showing the setup for injection of Cell Rovers into the cytoplasm of a Xenopus oocyte. The antenna is inserted into the membrane using a pair of tweezers and then pulled into the cell using a permanent magnet. The position of the magnet is controlled using a micromanipulator stage. **b** FEA simulation of magnetic field gradient around a permanent magnet (Neodymium N52 Bar Magnet with a 5 mm × 5 mm cross section). The dotted line shows the different positions of the cell during injection. **c** Magnetic field gradient $(\frac{\partial B}{\partial z})$ along the dotted line in **b** as a function of distance from the magnet surface. The range of optimum magnetic field gradient for successful injection is shown between the dotted lines. **d** Optical image of a Xenopus oocyte with a Cell Rover partially inserted into the cell membrane. **e** Optical image of the same cell in **c** after the antenna has been partially pulled in using the permanent magnet. **f** Optical image showing the site of injection after the antenna has been fully injected into the cell. **g** Zoomed in view of the site of injection shown in dotted lines in **f**. It can be seen that the membrane has fully healed after injection.

Cell Rovers were injected into Stage VI Xenopus Laevis oocytes (See Methods section for details on handling Xenopus oocytes) to study the antenna response from inside a cell. An injection scheme employing magnetic fields was developed to prevent leakage of cell cytoplasm and ensure cell viability after injection. Figure 3a shows a schematic diagram of the setup used for injection of Cell Rovers into Xenopus oocytes. The Cell Rover to be injected is sterilized using 70% ethanol and is partially inserted into the cell membrane using a pair of magnetic tweezers attached to a micromanipulator stage. The partially inserted antenna is pulled into the cell completely by applying a non-uniform magnetic field oriented in the vertical direction (z axis in Fig. 3a) using a permanent magnet attached to a micromanipulator stage. The force acting on the Cell Rover in the z-direction depends on the gradient of magnetic flux density ($\frac{\partial B}{\partial z}$) since it can be considered as a magnetic dipole in a non-uniform magnetic field (Force, ${{{{{\bf{F}}}}}}=\nabla ({{{{{\bf{m}}}}}}{{{{{\boldsymbol{.}}}}}}{{{{{\bf{B}}}}}})$, where ${{{{{\bf{m}}}}}}$ denotes the magnetic dipole moment and ${{{{{\bf{B}}}}}}$ denotes the magnetic flux density^29^). Note that the application of a high uniform magnetic field was not found to help in the injection.

To ensure effective intracellular injection the magnetic field gradient should have an optimal range, as a higher value is observed to cause the antenna to penetrate the cell membrane on the opposite side of the injection site (Supplementary Fig. 10) while a lower value is not sufficient to pull the antenna in. Figure 3b shows the distribution of the gradient of magnetic flux density $(\frac{\partial B}{\partial z})$ in the *z*–*x* plane around a permanent magnet of cross section 5 mm×5 mm and length of 4 cm used in the experiment, calculated using Comsol. The optimum magnetic field gradient required for successful injection was determined from multiple experiments by positioning the cell at different distances above the center of the permanent magnet after inserting the Cell Rover into the cell membrane. The dotted line in Fig. 3b shows the different positions of the cell and Fig. 3c shows the distribution of magnetic field gradient along this line as a function of the distance from the magnet surface. For distances <~3.5 mm away from the magnet surface, the Cell Rovers were found to penetrate the membrane on the side opposite to insertion indicating that the force is too high, whereas for distances >7.5 mm the force was found to be not high enough to pull the Cell Rovers into the cell. The optimum magnetic field gradient which ensures a successful injection as observed from the experiment and calculated using Comsol is shown between the dotted lines in Fig. 3c (75.0 G/mm < $(\frac{\partial B}{\partial z})_{{optimal}}$ < 315.0 G/mm). Figure 3d shows an optical image of a Cell Rover partially inserted into the cell membrane using the magnetic tweezers and Fig. 3e shows the same cell with the antenna slightly pulled in using the permanent magnet. Figure 3f, g shows an optical image of the site of injection 2 min after the antenna is fully pulled into the cell (See Supplementary Movie 2 for a video of the injection). It can be seen that the cell membrane has successfully healed after injection and the cell cytoplasm doesn’t leak due to the injection of the antenna. We further checked the cell viability after injection by observing the appearance of the two hemispheres of the cell membrane using optical imaging which is a standard viability assay for oocytes^30–33^, for 84 h as shown in Supplementary Fig. 11, and the cells were found to be viable (Supplementary Note 8).

### Wireless detection inside a living cell

**Fig. 4:**
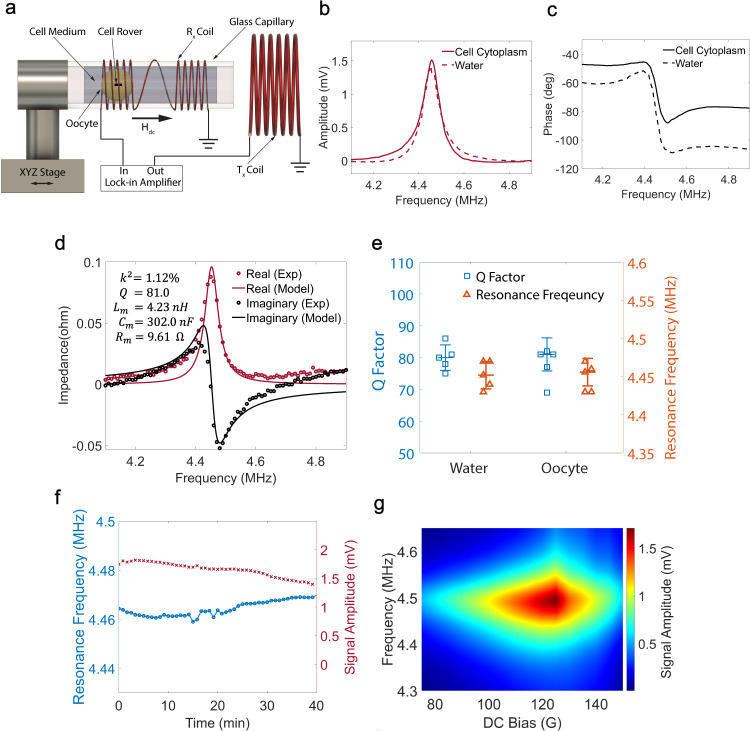
Wireless detection of Cell Rovers in Xenopus oocytes. **a** Schematic diagram showing the setup for wireless detection of a Cell Rover injected into a Xenopus oocyte. Measured **b** voltage amplitude and **c** phase of a Cell Rover wirelessly detected from inside the cell as a function of frequency of excitation magnetic field. The resonance frequency is found to be 4.456 MHz in cell cytoplasm. The dotted lines show the recorded response when the same resonator is in water. **d** Impedance vs Frequency of the Cell Rover inside the cell measured using a VNA and the corresponding equivalent circuit model fit which gives a mechanical quality factor (Q) of 81.0 and magnetomechanical coupling coefficient (${k}^{2}$) of 1.12%. The calculated values for motional inductance $({L}_{m})$, motional capacitance (${C}_{m}$), and motional resistance (${R}_{m}$) are also shown **e** Comparison of the resonance frequency and Q-factor of the Cell Rover in water and Xenopus oocyte cytoplasm. Plot shows individual points with bars showing median $\pm$ standard deviation for *n* = 5 samples each. **f** Variation of resonance frequency and signal amplitude of the Cell Rover inside a cell detected over time (40 min). **g** 2d plot showing the dependence of detected voltage amplitude on the DC bias field and frequency of excitation magnetic field. All measurements shown are for an intracellular antenna of dimensions 500 μm × 200 μm × 28 μm.

For measuring the antenna response from inside the cell, the Xenopus oocytes injected with Cell Rovers are inserted into the capillary tube holding the Rx coil along with 1X Modified Barth Saline (MBS) cell medium. The measurement setup is as shown in the schematic diagram in Fig. 4a. The setup is similar to Fig.1a but the capillary tube now contains the injected cell with the cell medium around it. The position of the cell inside the tube can be adjusted easily by flowing the cell medium through the capillary tube. All the results discussed in Fig. 4 are for a 500 μm × 200 μm × 28 μm intracellular antenna.

Figure 4b, c shows the detected coil voltage and phase respectively in cell cytoplasm, as a function of frequency of excitation magnetic field at the optimum bias field (125 Oe), with the dotted lines showing the detected response in water for the same resonator. Figure 4d shows the Impedance of the same intracellular antenna as a function of frequency, measured using a VNA. It has a resonance frequency of 4.456 MHz (standard deviation = 0.018 MHz, number of samples *n* = 5) and a Q-factor of 81.0 (standard deviation = 5.2, number of samples *n* = 5) when measured from inside the cell which is almost identical to that in water. It can be seen from Fig. 4d that the motional inductance and motional resistance values obtained from the equivalent circuit model in cell cytoplasm are also similar to the values obtained in water (Supplementary Table 2). Figure 4e shows the comparison between resonance frequency and Q-factor when the same Cell Rover is measured in water and cell cytoplasm for 5 different samples. From this data it can be seen that there is no significant difference between the resonator response in water and cell cytoplasm. These observations suggest that mass loading and viscous damping effects are very similar in both cases. The total coupling coefficient (${k}_{t}$) and wireless power transfer efficiency for operation in cell cytoplasm was also calculated to be 0.0066 and 0.63% respectively. Supplementary Fig. 7e shows the measured S11 response for the Tx coil containing the resonator in cell cytoplasm and the S11 response of the model for the best fit parameters. The resonator response in cell cytoplasm was also studied using analytical equations and the obtained values for mass loading and viscous damping parameters are shown in Supplementary Table 4. Supplementary Fig. 8c shows the simulated and measured response for the resonator vibration in cell cytoplasm.

For real time sensing experiments, it is important that the antenna has a stable frequency response with time. Several biologically relevant changes happen in Xenopus oocytes in the timescale of several minutes^34–37^. Hence, we measured the antenna response from inside the cell as a function of time for 40 min and recorded the resonance frequency and signal amplitude as shown in Fig. 4f. The measured resonance frequency has a mean of 4.46 MHz and a standard deviation of 3.09 kHz which makes it promising for real time sensing applications. It can also be seen that the amplitude remains stable with a mean of 1.65 mV and standard deviation of 0.12 mV. The near field coupling of the antenna from inside the cell is further studied by varying the DC bias magnetic field strength from 75 Oe to 150 Oe. Figure 4g shows the measured coil signal amplitude as a function of the DC bias and frequency of excitation. The detected signal has a maximum value of 1.7 mV at the optimum bias of 125 Oe for a 500 μm × 200 μm × 28 μm resonator. It reduces to 0.52 mV and 0.58 mV at bias fields of 75 Oe and 150 Oe respectively due to decrease in the magnetostrictivity of the material. This shows that applying an optimum bias field is critical for maximizing the signal amplitude and hence the signal to noise ratio of the resonator. Repeatability of the detected response from fabricated antennas of the same size is also critical for their real-world applications in sensing and stimulation. Variability in the detected response can arise from the random orientation of injected Cell Rovers inside the cell. We eliminate this by applying a DC magnetic field along the axis of the Rx coil which orients the Cell Rovers such that the length of the resonator is always along the axis of the Rx coil. Another source of variability is the small variations in sizes of the resonators during fabrication. To study this, we measured the frequency response from 8 different Cell Rovers of size 500 μm × 200 μm × 28 μm injected in eight different cells. They were found to have an average resonance frequency of 4.46 MHz with a standard deviation of 20.83 kHz and an average signal amplitude of 1.6 mV with a standard deviation of 0.1 mV which suggests that the fabrication process is repeatable and introduces minimal variation. Supplementary Fig. 12 shows voltage amplitude vs frequency plots for the 8 different resonators injected in different xenopus oocytes.

**Fig. 5:**
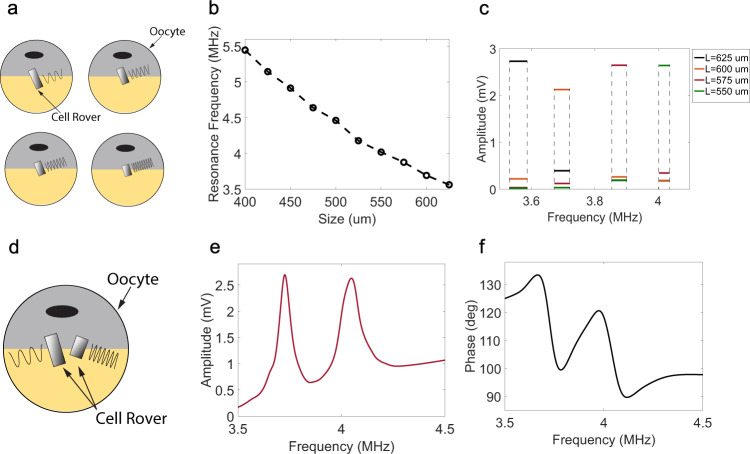
Multiplexing using Cell Rovers. **a** Schematic diagram showing different Xenopus oocytes injected with Cell Rovers of different sizes. Depending on the antenna size the resonance frequency of the signal detected from each oocyte will be unique. **b** Resonance frequency detected from different oocytes injected with different sized Cell Rovers as a function of their size. **c** Average detected voltage from cell rovers of different sizes for different frequency ranges. Each range shown by the dotted lines denotes the 3dB bandwidth of the resonator having the highest average signal in that range. **d** Schematic diagram showing a single Xenopus oocyte injected with two Cell Rovers, each one with its own characteristic resonance frequency. Wirelessly detected **e** voltage and **f** phase from a Xenopus oocyte injected with two Cell Rovers of sizes 600 μm × 200 μm × 28 μm and 550 μm × 200 μm × 28 um showing two distinct resonance frequencies.

Next, we check whether Cell Rovers can be used for multiplexing. Multiplexing is useful for intracellular applications because it can not only enable sensing or modulation of different cells using independent control signals but can also incorporate multiple functionalities in a single cell such as simultaneous sensing of more than one analyte within a cell. Cell Rovers with slightly different sizes can provide a pathway for both these applications since they have different resonance frequencies as given by Eq. (1). To test this, we fabricated 10 different sizes of Cell Rovers of cross section 200 μm × 28 μm but having lengths ranging from 400 μm to 625 μm. To check if different Xenopus oocytes can be addressed using signals of different frequency, these antennas were first injected into separate Xenopus oocytes as shown in the schematic diagram in Fig. 5a and their resonance frequency and amplitude was recorded. Figure 5b shows the recorded resonance frequency as a function of antenna length. Resonance frequency decreases with increase in size as expected from Eq. (1). Figure 5c shows the 3 dB bandwidth for Cell Rovers of different sizes and the detected coil voltage for the different Cell Rovers averaged over the range of frequencies in each bandwidth. It can be seen that the 3 dB bandwidths for different sized cell rovers do not overlap and compared to the antenna with the highest average signal in each bandwidth, the detected signal from the other antennas is negligible. This shows that different cells containing different sized Cell Rovers can be individually addressed using excitation magnetic fields at different frequencies. Next, to check if more than one antenna can be detected independently from the same cell, we injected a single Xenopus oocyte with Cell Rovers of two different sizes as shown in the schematic diagram in Fig. 5d. Figure 5e, f shows the amplitude and phase respectively, detected from an oocyte injected with Cell Rovers of lengths 550 μm and 600 μm and cross section 200 μm × 28 μm as a function of frequency of excitation magnetic field. The quality factor of the resonators is high enough such that two distinct resonance peaks which do not overlap, are observed, as can be seen in the frequency response. This shows that Cell Rovers can potentially be used for multiplexing applications within a single cell.

## Discussion

In this work, we have demonstrated Cell Rovers, a magnetostrictive intracellular antenna which can be wirelessly detected using near field inductive coupling from inside a Xenopus Laevis oocyte and operates at its mechanical resonance frequency when excited with an AC magnetic field. The frequency of operation is ~4.5 MHz for a resonator of dimensions 500 μm × 200 μm × 28 μm. This low MHz frequency is optimal for wireless operation in 3D biological systems without causing significant adverse heating effects^12, 14^. The Cell Rovers can be easily fabricated by micromachining of a 28 μm thick Metglas 2826 MB amorphous magnetostrictive film using a laser or diesaw. An injection scheme by applying a non-uniform magnetic field was developed to inject Cell Rovers into Xenopus Oocytes. They were then wirelessly detected from inside the cell and the frequency response was found to be similar to that in water. The effect of DC bias magnetic field on the detected voltage was also studied which showed that an optimum bias field is critical for obtaining maximum signal to noise ratio. The fabrication process was shown to be repeatable and any variability arising from the position of injected Cell Rovers was found to be minimized by orienting them using a DC magnetic field. Multiplexing using Cell Rovers of slightly different sizes was also successfully shown by injecting different oocytes with antennas of different sizes and also by injecting more than one antenna into the same oocyte.

Cell Rovers provide a versatile foundation stone upon which wireless sensing, modulation and in-cell computing capabilities can be built. Future work will involve configuring them to sense different intracellular entities such as inorganic ions (H^+^, Na^+^, K^+^, Cl^-^ etc) and biomolecules (Enzymes, RNA, DNA) important for cellular function as well as physical parameters like intracellular pressure and temperature. This can be achieved by functionalizing the resonators with reporters that produce a change in mass or stress in response to a change in the entity of interest^17^. The reporters can be for example, a polyacrylic acid polymer film which swells and shrinks in response to pH change^38^ or a biotinylated polyethylene glycol (PEG) film which can bind with avidin^39^. The resulting mass change in turn produces a change in resonance frequency of the resonator which can be detected wirelessly. For a change in mass (*Δm*) much smaller compared to the mass of the resonator (*M*), the resonance frequency shift is given by the below equation:

2 $$\triangle f=-\frac{f}{2}\frac{\triangle m}{M}$$

where, *f* is the original resonance frequency and Δ*f* is the change in resonance frequency^17^. Supplementary Fig. 13 shows the frequency shift as a function of mass change for a 500 μm × 200 μm × 28 μm resonator as given by Eq. (2). For a standard deviation of 3.09 kHz in the measured resonance frequency in cell cytoplasm, the minimum mass that can be detected using the developed technology is 29.4 ng (mass density of 14.7 µg/${{cm}}^{2}$). Much higher changes in mass density have been reported previously for magnetostrictive resonators designed for sensing ions^40^ and biomolecules^41^ at biologically relevant concentrations, which shows the feasibility of Cell Rovers to function as intracellular sensors. Since Cell Rovers of different sizes can be detected simultaneously from the same cell, we can enable simultaneous detection of multiple entities of interest from a single cell by functionalizing sensors of different sizes with different types of reporters. The frequency shift observed for resonators of different length would then correspond to different entities of interest. Functionalization of Cell Rovers with reporters might cause additional damping, but this will be negligible compared to damping by water and cell cytoplasm due to the low loss modulus of polymers and biomaterials used for functionalization. Supplementary Fig. 5c shows the simulated response from the analytical model of a resonator of dimensions 500 μm × 200 μm × 28 μm in cell cytoplasm with and without a 5 μm coating (20 μg total mass) of Polyethylene Glycol (PEG) having complex modulus^42^, ${E}_{f}^{*}=\left(10+j8\right){kPa}$, which is of the same order of magnitude as that for different polymers and biomaterials^42, 43^ (Supplementary Note 6). It can be seen that there is negligible change in the signal amplitude but a large change in the resonance frequency due to mass loading effects as reported previously for centimeter sized magnetoelastic resonators coated with paraffin^44^. This shows that large masses can be added to the resonator surface without causing a decrease in the measured signal amplitude. Apart from sensing, a piezoelectric layer can also be integrated onto the Cell Rover for converting the strain energy resulting from magnetic field excitation to electric field. This can be useful for energy harvesting for electrical stimulation as well as powering active nanoelectronic devices and circuits. Nanoelectronics can be fabricated on the Cell Rover platform leveraging the five decades of glorious Moore’s Scaling and advancement in Very Large Scale Integration (VLSI). This will enable the incorporation of the versatility of VLSI and nanoelectronic information processing within a living cell leading to unique smart functionalities not feasible with existing technologies.

Cell Rovers can be further miniaturized for application in smaller cells using conventional microfabrication techniques such as sputtering and photolithography. FeGaB magnetostrictive thin films (250–500 nm) with a magnetomechanical coupling coefficient as high as Metglas alloys have been grown using magnetron sputtering^45^. To investigate the feasibility of detection of Cell Rovers of smaller sizes, we have simulated using Comsol, the detected voltage and normalized detection range of a 25 μm × 25 μm × 5 μm magnetostrictive resonator using a modified Tx-Rx coil setup (Supplementary Note 9). Supplementary Fig. 14a shows the simulated response with a resonance frequency of 87.8 MHz and signal amplitude of 190 μV which is much higher than the noise level for detection (200 nV RMS). The detected signal amplitude as a function of *R/D* is shown in Supplementary Fig. 14b which gives a normalized detection range of 20.0, with a signal to noise ratio of 6.9 dB (Supplementary Note 7). This shows the feasibility of miniaturization of Cell Rovers. For such resonators operating at high frequencies eddy current losses in the resonator material are the dominating loss mechanism^46^. The effective magnetomechanical coupling efficiency (${k}_{{eff}}^{2}$) considering eddy current losses is given by the formula:^47^

3 $${k}_{{eff}}^{2}=\frac{{k}^{2}}{1+{\alpha }^{2}}$$

where ${k}^{2}$ is magnetomechanical coupling efficiency with zero eddy current losses and $\alpha$ is the eddy current loss angle which depends on conductivity ($\sigma$), permeability ($\mu$), thickness ($t$) of the magnetostrictive material and the operating frequency of the antenna ($\omega$) and is given by:

4 $$\alpha=\frac{\omega \sigma \mu {t}^{2}}{2}$$

Hence eddy current losses can be significantly minimized and the signal amplitude and detection range can be improved significantly compared to the simulation by depositing several thin magnetostrictive layers separated by insulating layers^47^.

Future work also involves expanding the applications to include in-vivo operation and Cell Rovers are particularly suited for this as they work with low frequency magnetic fields which have good tissue penetration^48^. For in-vivo operation, due to the conductivity of tissue there can be a loss in the measured signal amplitude as well as harmful heating effects due to the applied excitation magnetic field. However, since Cell Rovers operate using near field, low frequency magnetic fields both these effects are expected to be negligible. To show this, we have performed a CST simulation with an ideally exaggerated case of a muscle tissue medium (conductivity = 0.586 S/m at 4.5 MHz) with infinite boundary conditions, located 0.5 mm below the plane of the Tx coil. Supplementary Fig. 15a shows the simulated magnetic field strength vs distance from the Tx coil for air vs tissue which is observed to be almost identical. Hence there will be negligible loss in the excitation magnetic field as well as the detected signal when Cell Rovers operate in-vivo. The simulated Specific Absorption Rate (SAR) value in muscle tissue for the same case is as shown in the Supplementary Fig. 15b. The maximum SAR value is found to be 2.26 × 10−^4^ W/kg which is approximately four orders of magnitude below the accepted limits for local exposure to electromagnetic fields (2 W/kg^12^). This shows that the developed technology can be extended for in-vivo applications.

Thus, this technology has the potential to not only provide fundamentally new insights into biology, but also create novel pathways for health monitoring and therapeutics. Beyond healthcare, this fusion of nanotechnology and cells can lead to unique living hybrid robots, opening up myriads of interesting applications.

## Methods

### Antenna fabrication

Laser micromachining of Cell Rovers was performed using a 5 W Oxford 532 nm laser. The 28 μm thick film of Metglas 2826 MB was fixed on top of a 4-inch Silicon wafer using a permanent magnet fixture underneath the wafer. Cell Rovers of different sizes were machined at 1 % power, 20 kHz pulse frequency, 50 passes and a feed speed of 1 mm/s. Micromachining was also done on a DAD-3240 Diesaw with a 30-μm thick diamond blade. The 28-μm thick Metglas film was fixed on top of a 4-inch Silicon wafer using two layers of epoxy and Kapton tape to prevent the machined samples from being washed away by the cooling water jet. The assembly was cut at a feed speed of 3 mm/s and washed in acetone for 30 min, multiple times to release Cell Rovers of different sizes in solution. The released antennas were separated from solution using a permanent magnet and annealed at 220 °C for 2 h at 50 mTorr pressure to remove the residual stress in the material. See Supplementary Note 2 for more details on the fabrication.

### Coil design

The transmission (Tx) coil is made of 40 turns of 26 AWG wire, with a length of 17 mm and diameter of 6 mm to provide a uniform AC excitation magnetic field of 3 Oe. The lock-in amplifier output was connected to an RF amplifier (Texas Instruments THS 3092) which was then connected to the transmission coil to produce the required excitation field. The peak voltage and current through the coil were 8.0 V and 115.0 mA respectively at 4.5 MHz. Since the receiving (Rx) coil is inductively coupled to the Cell Rover, it is designed to have maximum number of turns, minimum parasitic capacitance and a self-resonance frequency (SRF) much higher than the frequency of interest which is ∼4.5 MHz for a 500 μm long resonator from Eq. (1). Each solenoid of the Rx coil was made of a single layer of 26 turns of 47 AWG wire with a length of 1 mm and diameter of 2 mm with an SRF of over 100 MHz. The diameter of the coil is also sufficiently greater than the cell diameter of 1.2 mm. The separation between the two solenoids was chosen to be 1 cm to prevent any coupling between them.

### Finite Element Analysis (FEA) of Cell Rover frequency response

The FEA modeling of Cell Rovers is done in Comsol Multiphysics using the Magnetostriction Module. The magnetostrictive material is modeled using linear piezomagnetic equations with an isotropic piezomagnetic coefficient or magnetostrictivity ($d$). A Rayleigh damping coefficient ($\alpha$) is introduced to account for the structural damping effects. The antenna is excited with a uniform AC magnetic field equal to the experimentally measured field and the amplitude and phase of the voltage across the Rx coil is simulated. The values of magnetostrictivity ($d$), Young’s modulus ($E$) and Rayleigh damping coefficient ($\alpha$) depend on the fabrication conditions and are chosen to best fit the experiment results with the simulated coil voltage amplitude. A detailed discussion of the Comsol simulation can be seen in Supplementary Note 3.

### Impedance measurement

A single solenoid of 2 mm diameter, 1 mm length and 26 turns made of 47 AWG wire was used for measuring the impedance of the resonator. The coil is wound on a 2 mm diameter tube and the resonator is positioned inside the coil using a permanent magnet for measurement. The coil was connected to an Agilent E5062A Vector Network Analyzer (VNA) and the S11 of the coil as a function of frequency was measured at 10 dBm power with and without the resonator present inside it. A permanent magnet was used to provide the optimum DC bias magnetic field. The port extension technique is utilized in the calibration step of the Vector Network Analyzer (VNA) to calibrate out the coaxial cable that was used for the S-parameter measurement. Since the cable length is significantly short compared to the wavelength at 4.5 MHz, the parasitic capacitance and resistance introduced in the cable are negligibly small, and therefore the calibration step is not necessary.

### Cell handling

Defolliculated Stage VI Xenopus Laevis Oocytes were purchased from Xenoocyte for all experiments. The cells were stored in a 16 °C incubator in 1X Modified Barth Saline with 0.1 mg/mL of pencillin-streptomycin as antibiotic prior to injection. The Agarose gel plate for injection is prepared by making a 2% solution of Agarose in sterilized Micropure water. The solution is microwaved for 30 s to ensure complete dissolution. The cells along with the cell medium is then transferred to the gel plate for injection. After injection the cells are again transferred to 1X MBS medium and stored at 16 °C for optical imaging. The cell medium is changed every few hours to prevent bacterial growth. All our experiments complied with all relevant ethical regulations and were approved by the Institutional Animal Care and Use Committee at Massachusetts Institute of Technology.

## Supplementary information

Supplementary Information

Description of Additional Supplementary Files

Supplementary Movie 1

Supplementary Movie 2

## Data Availability

The authors declare that the data supporting the findings of this study are available within the paper and its Supplementary Information file. Source data are provided with this paper.

## Source data

Source Data
