# Supplementary material for: Cell Rover—a miniaturized magnetostrictive antenna for wireless operation inside living cells
Source: Nat Commun. 2022 Sep 22;13:5210. doi: 10.1038/s41467-022-32862-4 (PMC9499948; doi:10.1038/s41467-022-32862-4)
Supplement: Supplementary file 1 — Supplementary Information [file 41467_2022_32862_MOESM1_ESM.pdf]

Supplementary Information

Cell Rover – A Miniaturized Magnetostrictive Antenna for Wireless Operation Inside Living Cells

Joy et al.

Supplementary Note 1: Principle of Magnetostriction

Magnetostrictive behaviour can be observed in materials which exhibit strong spin-orbit coupling which causes these materials to experience a strain or magnetostriction (λ) in response to magnetization (Joule Effect) and vice-versa (Villari Effect). Even though the magnetostrictive behaviour of a material is non-linear with respect to applied magnetic field, for an amorphous magnetic material at a certain DC magnetic field bias it can be approximated by using piezomagnetic equations with an isotropic piezomagnetic coefficient or magnetostricitivity (d). Supplementary Fig. 1a shows a schematic representation of the nonlinear dependence of magnetostriction (λ) of a material on the applied magnetic field strength (H). The magnetostrictivity ($d = \frac{d\lambda}{dH}$) is given by the slope of the curve at the bias point and has a maximum value when $H = H_{dc,opt}$ as illustrated in Supplementary Fig. 1b. When a thin film mechanical resonator made of such a material is biased and excited with a small AC magnetic field, it generates acoustic waves in the material and there is a resonance in the strain and magnetization in the material at the natural frequency of vibration of the resonator. Hence, it functions as a strain powered antenna which radiates efficiently at its mechanical resonance frequency in contrast to the much higher electromagnetic resonance frequencies of conventional antennas. The radiation performance of a magnetostrictive antenna depends on the magnetomechanical coupling coefficient (k^2) of the material, structural and viscous damping, and dimensions of the antenna. The magnetomechanical coupling coefficient denotes the efficiency of conversion of input magnetic energy to mechanical energy and is given by:

$$k^2 = \frac{Stored\ Mechanical\ Energy}{Input\ Magnetic\ Energy} = \frac{\frac{1}{2}\frac{(dH)^2}{s}}{\frac{1}{2}\mu H^2} = \frac{d^2}{\mu s} \qquad (1)$$

where μ is the relative permeability and s is the compliance coefficient of the material. We have chosen Metglas 2826 MB which has a high reported magnetomechanical coupling

coefficient compared to other magnetostrictive materials. Coupling coefficient values as high as k=0.9-0.98 have been reported previously for Metglas alloys, measured for centimetre sized resonators operating at frequencies of 10s of kHz[1,2]. As the operating frequency increases the magnetomechanical coupling efficiency decreases due to eddy current losses in the resonator material.

Supplementary Note 2: Fabrication of Cell Rovers

We have explored two different methods for fabricating Cell Rovers from a 28 µm thick film of Metglas 2826 MB amorphous magnetostrictive material. The frequency response of the fabricated antennas was found to be identical for both methods. The first method involves Laser Micromachining of the film using a 5W Oxford 532 nm laser. The setup for laser micromachining is as shown in Supplementary Fig. 2a. The film is fixed on a 4-inch Silicon wafer using a permanent magnet fixture taped to the bottom of the wafer. The Silicon wafer acts as a good conductor to prevent excessive heating of the sample during laser micromachining. Excessive heating causes the material to become brittle and lose its magnetostrictive properties. The permanent magnet ensures that the thin film material is held flat and does not bow during the machining and also ensures good thermal contact between the film and the wafer. The magnet is fixed to a movable stage which can be programmed to cut Cell Rovers of different sizes. The cut antennas can be separated easily by using the permanent magnet after removing the remainder of the film. The laser machining parameters were optimized to ensure the best resonator response. A laser power of 1%, pulse frequency of 20 kHz, feed speed of 1 mm/s and 50 passes was found to be ideal for Cell Rovers of sizes of the range 500 µm x 200 µm. Laser micromachining has the advantage that it doesn't require any prior sample preparation or post processing annealing and are ideal for cutting round edges and shapes other than rectangles.

The second method involves micromachining of the film using a DAD-3240 Diesaw with a 30-µm thick diamond blade. Supplementary Fig. 2b shows the steps involved in the process. The Metglas film is first fixed to a 4-inch Silicon wafer using two layers of temperature

curable epoxy and Kapton tape to ensure that the diced resonators are not washed away during machining. After making the cuts at a feed speed of 3 mm/s the sample is ultrasonicated in acetone for 30 minutes multiple times to remove the epoxy and antennas of different sizes are collected using a permanent magnet as shown in Supplementary Fig. 2b. They are then annealed at 220 °C at 50 mTorr pressure in a vacuum oven to remove any residual stresses. Diesaw was found to have a better throughput but is ideal only to fabricate rectangular shaped antennas and involves longer sample preparation and post processing steps compared to laser micromachining.

Supplementary Fig. 2c and 2d shows the optical images of Cell Rovers of size 500 µm x 200 µm x 28 µm fabricated using Laser micromachining and Diesaw respectively. It can be observed that Laser micromachining produces rougher edges compared to Diesaw because of the finite spot size of the laser (5 µm) which melts the material. However, no difference was observed in the detected response from resonators fabricated using the two different methods. Supplementary Fig. 2e shows the detected response from two resonators of size 500 µm x 200 µm x 28 µm fabricated using Laser micromachining and Diesaw which is observed to be almost identical.

Supplementary Note 3: Comsol Simulation

The frequency response of Cell Rovers is modeled using the Magnetostriction module in Comsol Multiphysics which couples the AC/DC module and Structural Mechanics module. Due to lack of any 2D symmetry the modelling is done in 3D domain. The magnetostrictive material at optimum DC bias magnetic field is characterized by the following linear piezomagnetic equations using magnetostrictivity (d), Young's Modulus (E), and Poisson's ratio (ν) of the material:

$$\varepsilon = s_H S + {d_{HT}}^T \boldsymbol{H} \tag{2}$$

$$\boldsymbol{B} = d_{HT} S + \mu_0 \mu_r \boldsymbol{H} \tag{3}$$

where ε, S, $\boldsymbol{H}$, and $\boldsymbol{B}$ denote the strain, stress, magnetic field and magnetic flux density, μ_0 is the magnetic permeability of free space, μ_r is the relative permeability at constant stress, and s_H, d_{HT} denote the compliance matrix and piezomagnetic coupling matrix which is as given below for an isotropic magnetostrictive material:

$$d_{HT} = \begin{bmatrix} d & 0 & 0 & 0 & 0 & 0 \\ 0 & d & 0 & 0 & 0 & 0 \\ 0 & 0 & d & 0 & 0 & 0 \end{bmatrix} \tag{4}$$

$$s_H = \begin{bmatrix} 1/E & -\nu/E & -\nu/E & 0 & 0 & 0 \\ -\nu/E & 1/E & -\nu/E & 0 & 0 & 0 \\ -\nu/E & -\nu/E & 1/E & 0 & 0 & 0 \\ 0 & 0 & 0 & (1+\nu)/E & 0 & 0 \\ 0 & 0 & 0 & 0 & (1+\nu)/E & 0 \\ 0 & 0 & 0 & 0 & 0 & (1+\nu)/E \end{bmatrix} \tag{5}$$

A Rayleigh damping factor (α) is introduced to account for structural damping effects. A fixed boundary condition is assigned to the mid-plane of the resonator to account for it being the nodal plane and all the other faces are assigned as free. The antenna is placed in a large air domain to model the magnetic flux density around the antenna using Ampere's law. The boundaries of the air domain are magnetically insulated to prevent any flux leakage. The antenna is also surrounded by the gradiometer coil of same parameters as used in the experiment to model the inductive coupling. The coil is modelled as a homogenized current-carrying domain with 26 turns of 47 AWG wire. A uniform AC magnetic field of 3 Oe directed along the length of the antenna, equal to the experimentally applied magnetic field is used for exciting the magnetostrictive material and the frequency of the excitation field is swept to simulate the frequency response. The values of parameters d, E, and μ_r depends on the fabrication conditions and are estimated by matching the simulation results with the experimental data. The material properties used in the simulation are as shown in Supplementary Table 1. Supplementary Fig. 3 shows the simulated tip displacement of the resonator as a function of frequency of excitation magnetic field. The resonance frequency in air is shown to be 4.532 MHz. Supplementary Movie 1 shows the time domain response of the resonator at the resonance frequency with the deformation scaled by a factor of 2000X.

Supplementary Note 4: Range of detection

Normalized detection range of a near field antenna is defined as given below:

$$\frac{R}{D} = \frac{Distance\ between\ antenna\ and\ receiver}{Effective\ Size\ of\ antenna} \tag{6}$$

The measured signal amplitude for Cell Rovers of size 500 μm x 200 μm as a function of distance from the receiving coil are as shown in Supplementary Fig. 4. The noise power spectral density at the input of the lock-in amplifier was measured to be 20 nV/$\sqrt{\mathrm{Hz}}$. Measurements were done using a 100 Hz bandwidth for the low pass filter and 10 mV range on the Zurich Instruments lock-in amplifier which gives a noise level of 200 nV RMS which is also shown in Supplementary Fig. 4. Due to operation at 4.5 MHz Cell Rovers are more suitable for near field inductive coupling at large distances and it can be detected with a signal to noise ratio (SNR) of 19.5 dB (Signal amplitude = 1.9 μV) at a distance of 1 cm outside the receiving coil. At 1 cm distance for a 500 μm x 200 μm resonator having an effective diameter (diameter of a circle having the same area as the resonator) of 357 μm we get an R/D value of 28.0.

Supplementary Note 5: Equivalent Circuit Model

The equivalent circuit modeling of the resonator is done as shown in Reference [1][3]. The equivalent circuit is as shown in Supplementary Fig. 5. L_c (=270 nH), R_c (=1.9 Ω) denotes the inductance and resistance of the Tx coil of diameter 2 mm, length 1 mm and 26 turns at 4.5 MHz frequency calculated from the S11 data of the Tx coil without the resonator. L_r denotes the inductance of the antenna arising from the constant stress permeability (μ_r) of the resonator material. k^2 denotes the magnetomechanical coupling coefficient which is the ratio of stored mechanical energy to the input magnetic energy. The resonator impedance is represented by a parallel RLC circuit. L_m, R_m, C_m denote the motional inductance, motional resistance and motional capacitance of the resonator which depend on the mass, damping

and stiffness respectively[3]. At the resonance frequency ($\omega_0 = \frac{1}{\sqrt{L_m C_m}}$) the imaginary part of the impedance is zero and the real part is maximum. The value for the different parameters can be obtained by fitting the experimental S11 data to that calculated for the model. The expressions for the resonator impedance parameters are as given below:

$$L_m = L_r \frac{8}{\pi^2} \tag{7}$$

$$C_m = \frac{1}{\omega_0^2 L_m} \tag{8}$$

$$R_m = Q\omega_0 L_r \frac{8}{\pi^2} \tag{9}$$

where Q, $\frac{\omega_0}{2\pi}$ are the mechanical quality factor and resonance frequency respectively. The quality factor Q denotes the ratio of energy stored in a cycle to the energy dissipated due to damping. The equivalent circuit component values are as shown in Supplementary Table S2 for a 500 µm x 200 µm x 28 µm resonator in air, water and cell cytoplasm.

Supplementary Note 6: Wireless Power Transfer Efficiency

Unlike conventional electromagnetic antennas magnetostrictive antennas do not convert electromagnetic energy into current and voltage, but instead into strain energy. Therefore, the conventional two-port S-parameter analysis on the wireless power transfer system[4] is not possible for the passive Cell Rover device. Alternatively, a magnetostrictive antenna can be modeled equivalently as a magnetic dipole or an analytically infinitesimal solenoid of the same length and cross-sectional area along the in-plane direction of the piezomagnetic layer[5]. The inductive coupling between the transmitting coil and the magnetostrictive antenna, as well as the power transfer efficiency, can be further extracted from the model by measuring the reflection coefficient of the transmitting coil, with and without the presence of the antenna using a VNA. We can derive the equivalent model in a circuit schematic as shown in Supplementary Fig. 7a. R_s is the source resistance of the characterization equipment. L_1 and R_1 are the inductance and resistance of the transmitting (Tx) coil, which can be measured directly from impedance measurement of the Tx coil. L_2 , C_2

and R_2 are the inductance, capacitance and resistance of the resonant circuit which models the magnetostrictive antenna. Quality factor of the antenna characterizes the ratio of the stored energy over the dissipated energy and therefore is equal to $\omega L_2/R_2$, where ω is the angular frequency. L_1 and L_2 are coupled by a total coupling coefficient of k_t and their mutual inductance (M_{12}) is given as,

$$M_{12} = k_t\sqrt{L_1 L_2} \tag{10}$$

The input impedance Z_{in} of the transmitting coil in the presence of the Cell Rover can be found to be[6],

$$Z_{in} = R_1 + k_t^2(\omega L_1)\frac{Q}{1+Q^2\left(1-\frac{\omega_0^2}{\omega^2}\right)^2} + j\left[\omega L_1 - k_t^2(\omega L_1)\frac{Q^2\left(1-\frac{\omega_0^2}{\omega^2}\right)}{1+Q^2\left(1-\frac{\omega_0^2}{\omega^2}\right)^2}\right] \tag{11}$$

where k_t is the total coupling coefficient between the two antennas, Q is the quality factor of the magnetostrictive antenna, and ω_0 is the mechanical resonance frequency. The reflection coefficient S_{11} can be further calculated as,

$$S_{11} = \frac{Z_{in} - R_s}{Z_{in} + R_s} \tag{12}$$

A single solenoid of 2 mm diameter, 1 mm length and 26 turns made of 47 AWG wire was used for measuring the reflection coefficient and it is the same solenoid used for the resonator impedance measurement detailed in the methods section. By fitting the experimental reflection coefficient data, we can find the Q factor and the total coupling coefficient to be 497.0 and 0.0066 respectively, as shown in Supplementary Fig. 11b. Supplementary Fig. 7c and 7d demonstrate the variance in the output of the model if the total coupling coefficient or the quality factor is altered from the best fit parameters. Finally, assuming maximum power transfer condition is satisfied and $Q_{coil} = 4$ for the receiving coil, the equivalent power transfer efficiency of the Cell Rover in air is given by the equation[7],

$$\eta_{opt} = \frac{k_t^2 Q_{coil} Q}{\left(1 + \sqrt{1 + k_t^2 Q_{coil} Q}\right)^2} \quad (13)$$

This comes out to be 3.67% for resonator vibration in air.

Similar measurements were done for Cell Rovers in water and cell cytoplasm and the experimental and simulated reflection coefficients of the Tx coil are as shown in Supplementary Fig. 7e and Fig. 7f respectively. The power transfer efficiency, quality factor and coupling coefficients obtained for all three cases are shown in Supplementary Table 3.

Supplementary Note 7: Analytical Modelling

The frequency response of Cell Rovers was also modelled using one-dimensional analytical equations to understand the viscous damping and mass loading effects due to operation in water and cell cytoplasm as well as due to functionalization of reporters on the resonator surface. We consider a Cell Rover of length $2L$, width w and thickness t_s, Young's modulus E, piezomagnetic coefficient d and permeability μ_r with length along the x-axis and centered at the origin. The 3D constitutive equations as described by equation (2) and equation (3) are reduced into 1D equations by assuming that the stresses and strains are confined to the longitudinal direction of the resonator. Equations (2) and (3) can be simplified to obtain:

$$\varepsilon_{xx} = \frac{\sigma_{xx}}{E} + dH_x \quad (14)$$

$$B_x = d\sigma_{xx} + \mu_0 \mu_r H_x \quad (15)$$

where ε_{xx}, σ_{xx}, H_x, B_x, and H_x denote the normal strain, normal stress, magnetic field and magnetic flux density in the x-direction. The governing equation is derived by applying Newton's second law to an infinitesimal section of the resonator as shown in Supplementary Fig. 8a. The resonator is functionalized on both sides with a polymer film of thickness t_f, density ρ_f, and complex modulus E_f^*. The strain in the polymer film is assumed to be same as that of the resonator. A thin layer of liquid above it denotes the liquid layer that is activated by

the motion of the resonator and is represented by the mass loading term m_w. The viscous damping coefficient due to the liquid is denoted by c_{visc}. The value of c_{visc} and m_w depends on the viscosity of the liquid, frequency of excitation and distance between the resonator surface and the stationary boundary (Cell membrane or wall of the capillary tube)[8] and is difficult to estimate analytically for a freestanding resonator resting on a stationary surface. The structural damping force F_{struc} is modelled by considering Rayleigh damping with a damping parameter (α). For a resonator of mass *m*, density *ρ* and cross-sectional area *A*, F_{struc} can be written as:

$$F_{struc} = \alpha m = \alpha \rho A \tag{16}$$

The displacement of the infinitesimal mass of the resonator is denoted by the function $u(x,t)$, where:

$$\varepsilon_{xx} = \frac{\partial u}{\partial x} \tag{17}$$

The simplified governing equations for the vibration of the resonator shown in Supplementary Fig. 8a are as given below:

$$\begin{aligned}\left[\left(E_{app} - \frac{d'^2 {E_{app}}^2}{\mu_0 \mu_r'}\right) t_s + 2E_f^* t_f\right] \frac{\partial^2 u}{\partial x^2} - \left[\rho t_s + m_w + 2\rho_f t_f\right] \frac{\partial^2 u}{\partial t^2} \\ - \left[c_{visc} + \alpha \rho t_s\right] \frac{\partial u}{\partial t} \quad = \quad E_{app} d t_s \frac{\partial H_x}{\partial x}\end{aligned} \tag{18}$$

where

$$E_{app} = \frac{E \mu_0 \mu_r}{\mu_0 \mu_r - d^2 E} \tag{19}$$

$$\mu_0 \mu_r' = \mu_0 \mu_r - d^2 E \tag{20}$$

$$d' = \frac{d(\mu_0 \mu_r - d^2 E)}{\mu_0 \mu_r} \tag{21}$$

which can be solved using eigenfunction expansion to obtain the solution for $u(x,t)$ as described in Ref. [9] [9]. The voltage and phase of the coil wrapped around the resonator is

given by the rate of change of magnetic flux density inside the coil. The resonator is assumed to be a magnetic dipole of length $2L$ to estimate the magnetic flux density distribution in the region around the resonator. Supplementary Fig. 8b shows the comparison between the measured resonator response in air and water to that simulated from the analytical model when the polymer film thickness is zero. Material parameters used are same as that in Supplementary Table 1. Supplementary Fig. 8c shows the comparison between measured and simulated resonator response in cell cytoplasm. The values for c_{visc} and m_w obtained from the simulation are shown in Supplementary Table 4 for measurement in water and oocyte and can be seen to be almost similar. The mass loading and viscous damping effects due to air is assumed to be negligible.

To understand the damping effects that would be caused by functionalizing the resonator, we assume that the resonator is coated with a 5-μm film of polyethylene glycol (PEG) of complex modulus[10], $E_f^* = (10 + j8)\ kPa$, which is of the same order of magnitude as that for different polymers and biomaterials[11]. Supplementary Fig. 10d shows the simulated resonator response in cell cytoplasm with and without the PEG film. It can be seen that due to the low loss modulus of the polymer, there is negligible change in the signal amplitude but a large change in the resonance frequency of the resonator due to mass loading.

Supplementary Note 8: Cell Viability

The most common method of checking cell viability in Xenopus oocytes is by optically imaging the morphological features of oocytes[12–15]. Stage VI Xenopus oocytes are viable if the cell has distinct brown and pale-yellow hemispheres, with a narrow unpigmented equatorial band[16] as shown in Supplementary Fig. 11a. Dead oocytes are characterized by uneven pigmentation as shown in Supplementary Fig. 11b. Supplementary Fig. 11c, 11d, 11e, 11f, 11g, 11h, 11i, 11j shows the optical images of the cell at times, t = 1 hr, 2 hr, 4 hr and 8 hr, 12 hr, 24 hr, 48 hr and 84 hr after injection. The appearance of the injected cells clearly shows that the Xenopus oocytes are viable for up to 84 hours after injection. When stored in 1X MBS

solution with proper antibiotics and daily changes in medium normal Xenopus oocytes generally survive for about 3-4 days after defolliculation[15] (2-3 days after injection, since shipping of defolliculated oocytes takes 1 day). Since the cells were found to be viable for up to 84 hr after injection, we have concluded that the cells are not adversely affected by the injection of Cell Rovers.

Supplementary Note 9: Miniaturization of Cell Rovers

To investigate the minimum size of Cell Rovers which can be detected using Tx and Rx coils, we have simulated using Comsol, the detected voltage from a 25 μm x 25 μm x 5 μm magnetostrictive resonator. The material parameters are same as that obtained from the experiment for the 500 μm x 200 μm x 28 μm resonator shown in Supplementary Table 1. The Rx coil has been modified for smaller resonators and consists of two solenoids with a length and diameter of 100 μm and 60 turns each. The excitation magnetic field applied is maintained at 3 Oe. Supplementary Fig. 14a shows the simulated response with a resonance frequency of 87.8 MHz and signal amplitude of 190 μV which is much higher than the noise level for detection (200 nV RMS). The normalized detection range of the resonator was also determined using the simulation by varying the position of the resonator along the axis of the coil and the signal amplitude obtained as a function of *R/D* is as shown in Supplementary Fig. 14b. It can be seen from the figure that a normalized detection range of 20.0 with a signal to noise ratio of 6.9 dB (Signal Magnitude = 0.45 μV) can be obtained using the proposed Tx-Rx coils.

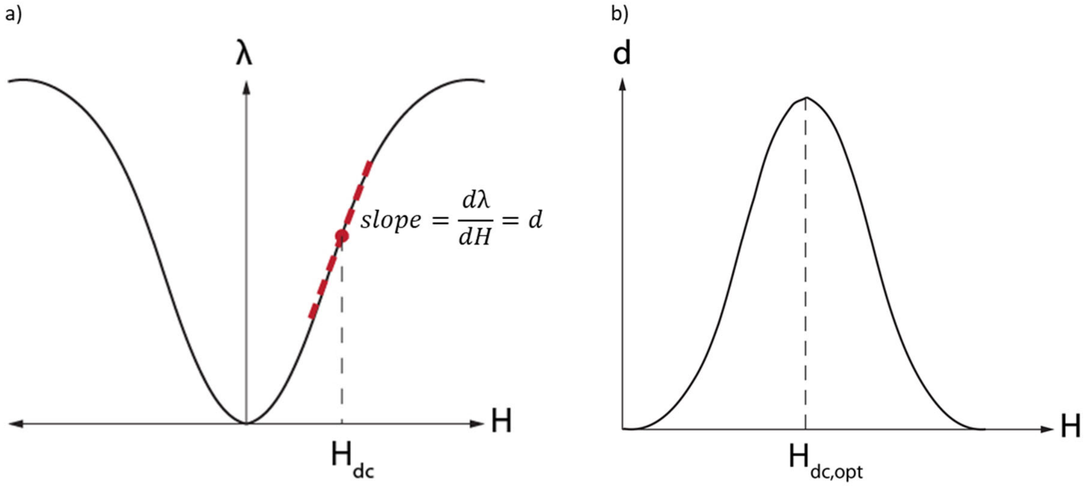

Supplementary Fig. 1. Schematic representation of magnetostriction and magnetostrictivity. a) Schematic diagram showing the magnetostriction (λ) of a material as a function of applied magnetic field strength (H). Magnetostrictivity (d) of the material at DC bias field (H_{dc}) is given by the slope of the curve at the bias point. **b)** Schematic diagram showing the magnetostrictivity (d) of a material as a function of applied magnetic field strength (H). The optimum DC magnetic field which maximizes the magnetostrictivity is shown as $H_{dc,opt}$.

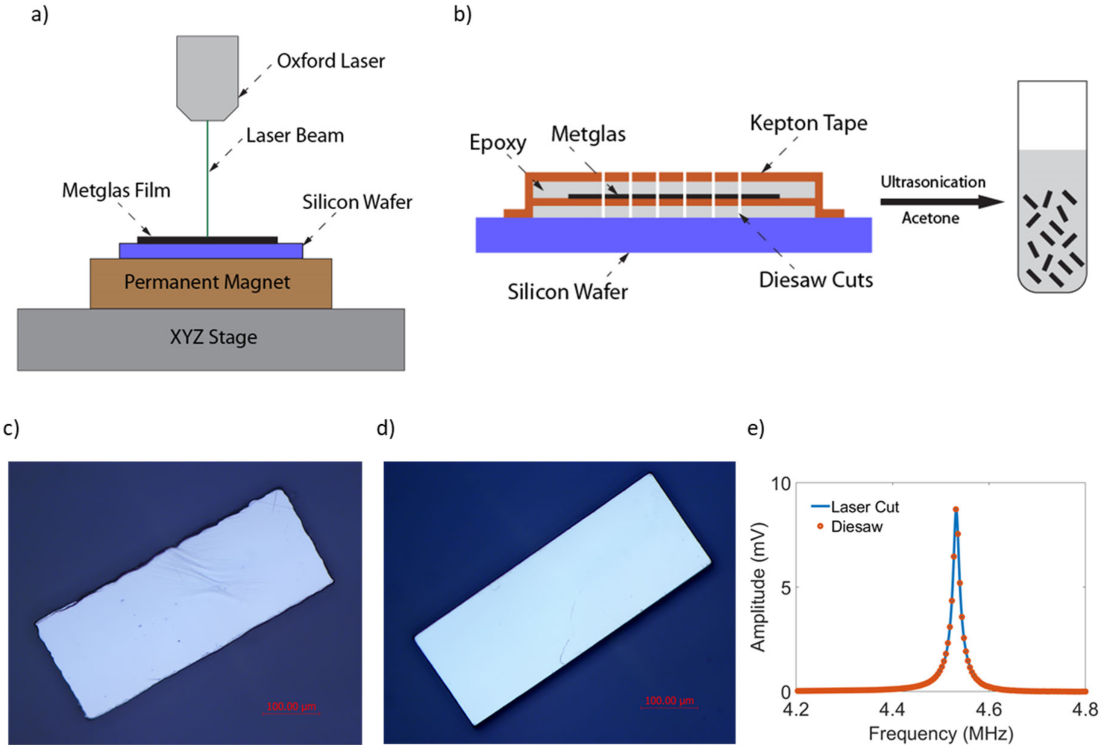

Supplementary Fig. 2. Fabrication of Cell Rovers. (a) Schematic diagram showing the setup for the fabrication of Cell Rovers by laser micromachining using an Oxford 532 nm Laser. **(b)** Schematic diagram showing the steps involved in the fabrication of Cell Rovers by micromachining using a Diesaw. Optical images of Cell Rovers of dimensions 500 µm x 200 µm x 28 µm fabricated using **(c)** Laser micromachining and **(d)** Diesaw respectively. **(e)** Comparison of measured signal amplitude as a function of frequency of excitation magnetic field (3 Oe) for Cell Rovers of dimensions 500 µm x 200 µm x 28 µm fabricated by Laser micromachining and Diesaw.

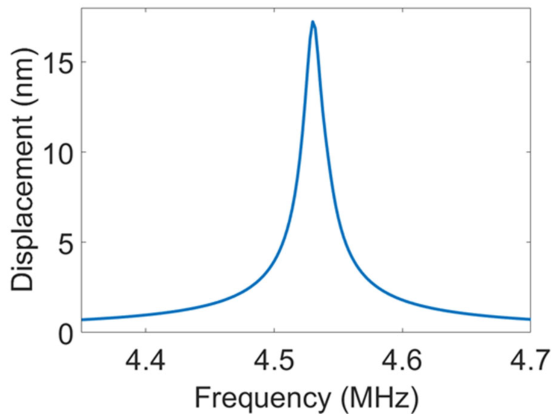

Supplementary Fig. 3. Comsol simulation of resonator displacement. Comsol simulation of the displacement at the tip of a 500 µm x 200 µm x 28 µm resonator as a function of frequency of the excitation magnetic field of amplitude 3 Oe. The resonance frequency is found to be 4.532 MHz.

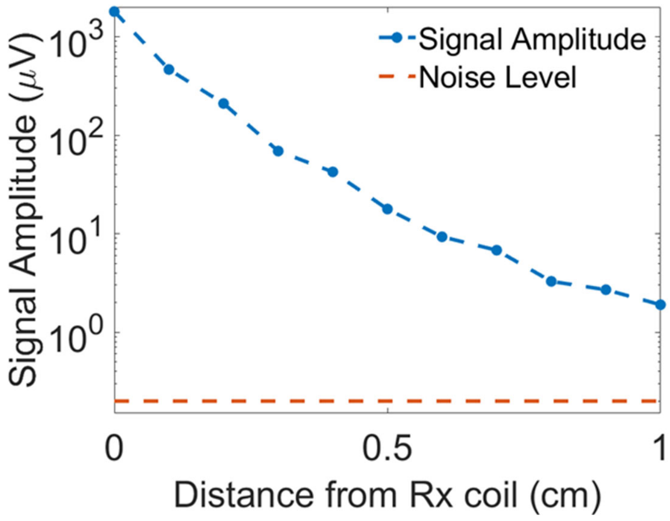

Supplementary Fig. 4. Detection of Cell Rovers outside the Rx coil. Wirelessly detected signal amplitude from a Cell Rover of dimensions 500 µm x 200 µm x 28 µm as a function of the distance from the Receiving coil. The noise level (200 nV) is also shown**.**

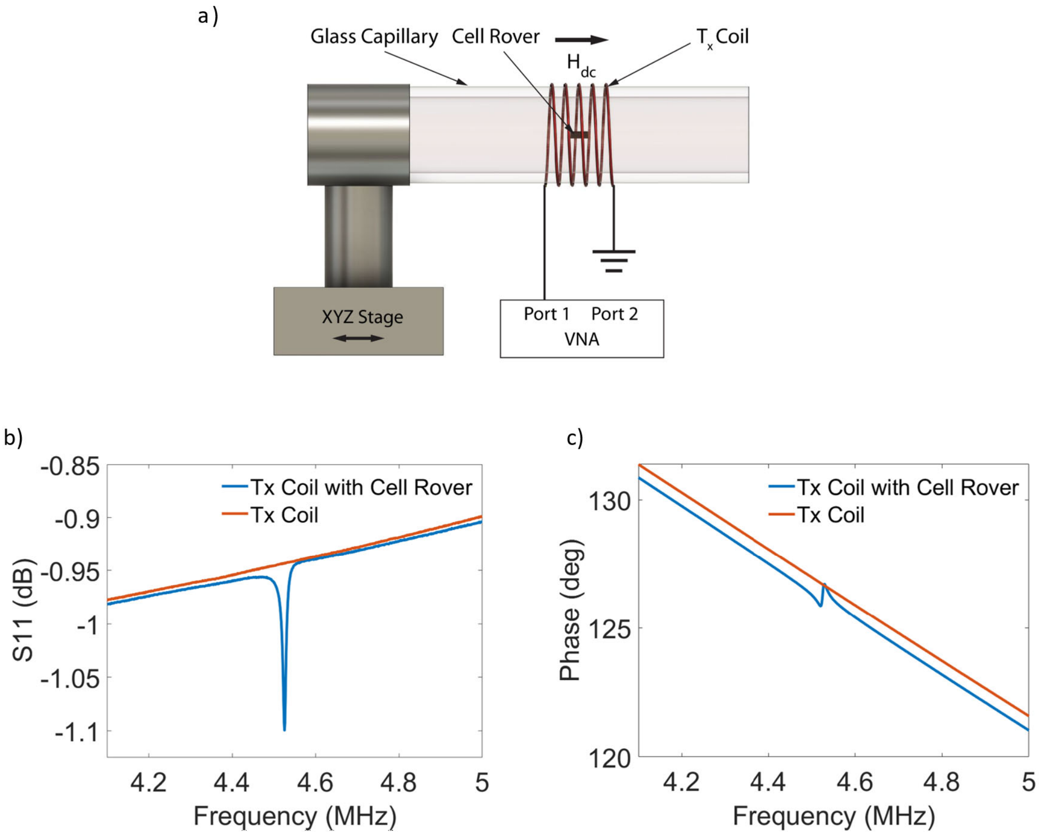

Supplementary Fig. 5. Cell Rover characterization using VNA. a) Schematic diagram showing the setup used for impedance and power transfer characterization of Cell Rovers which consists of a single solenoid of 2 mm diameter, 1 mm length and 26 turns connected to a Vector Network Analyzer (VNA). Measured S11 **(b)** magnitude and **(c)** phase for the Tx coil with and without the resonator inside it as a function of frequency.

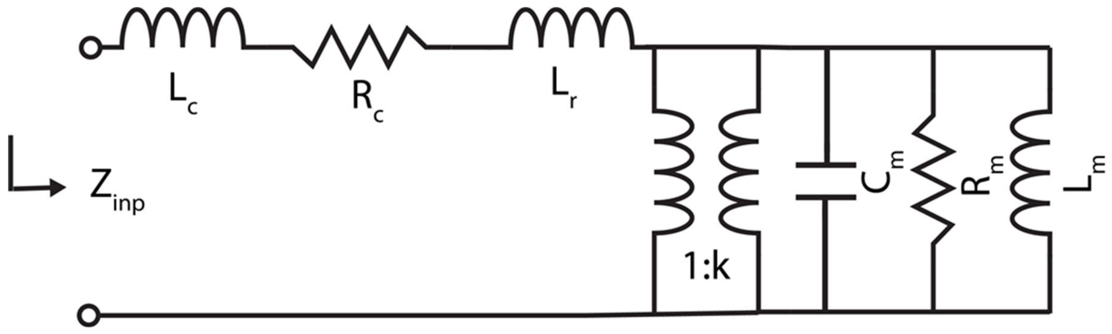

Supplementary Fig. 6. Equivalent circuit model of a magnetostrictive resonator coupled with a Tx coil.

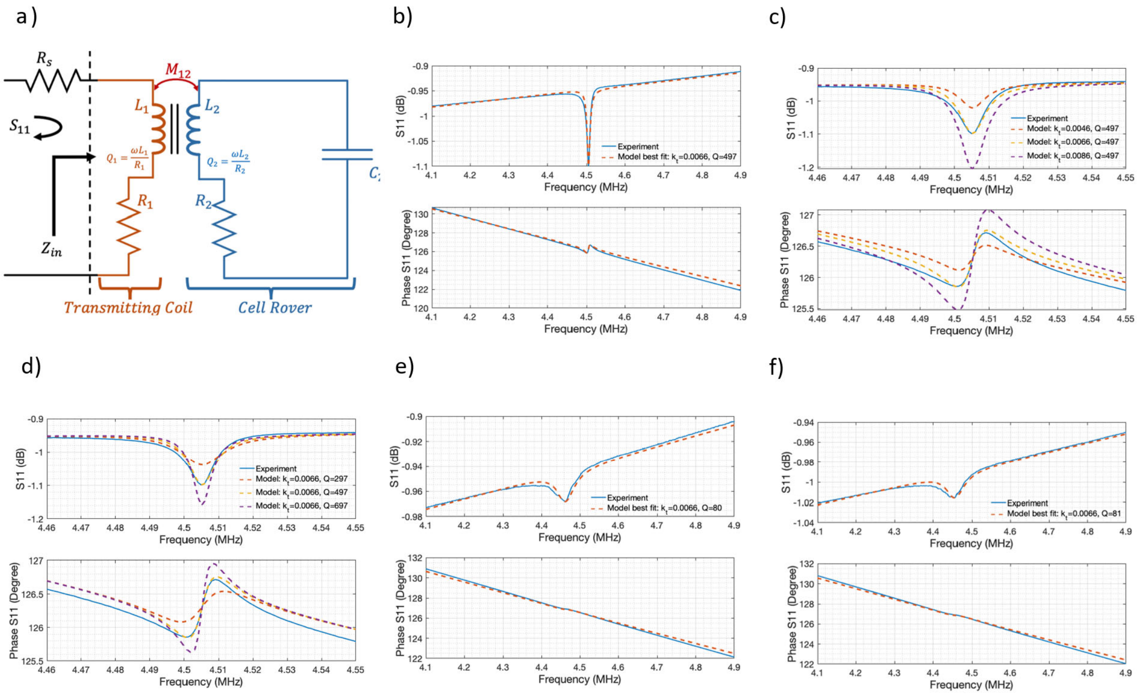

Supplementary Fig. 7. Coupling coefficient and wireless power transfer efficiency of Cell Rovers (a) Equivalent circuit model of a magnetostrictive antenna coupled to a transmitting coil modelled as two inductively coupled solenoids. **(b)** Comparison between measured and best fit S11 magnitude and phase for a 500 µm x 200 µm x 28 µm resonator coupled to a Tx coil of diameter 2 mm, length 1 mm and 26 turns. Variation in the calculated S11 for different values of **(c)** total coupling coefficient and **(d)** quality factor respectively showing the accuracy of the proposed model. Comparison between measured and best fit S11 magnitude and phase for a 500 µm x 200 µm x 28 µm resonator coupled to a Tx coil of diameter 2 mm, length 1 mm and 26 turns in **(e)** water and **(f)** cell cytoplasm respectively.

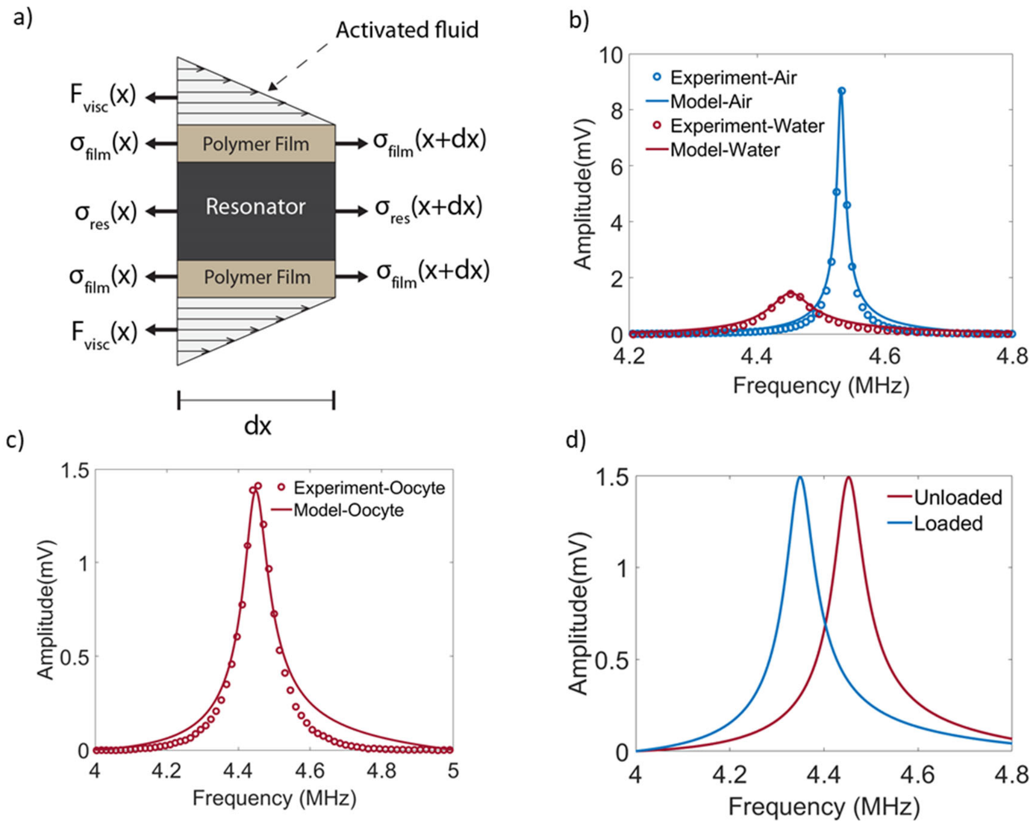

Supplementary Fig. 8. Analytical modelling of Cell Rovers. (a) Schematic diagram showing the different forces acting on an infinitesimal mass of the resonator in a liquid with a polymer coated on both sides. The approximate velocity profile of the activated fluid is also shown. In addition to these forces the resonator also experiences structural damping forces. **(b)** Signal amplitude measured from experiment and that calculated from the analytical model for a resonator of dimensions 500 μm x 200 μm x 28 μm in air and water as a function of frequency of excitation magnetic field (3 Oe). **(c)** Signal amplitude measured from experiment and that calculated from the analytical model for the same resonator in cell cytoplasm as a function of frequency of excitation magnetic field (3 Oe). **(d)** Signal amplitude vs frequency calculated from analytical model for the same resonator in cell cytoplasm with and without loading by 5 μm thick layer of PEG on both sides.

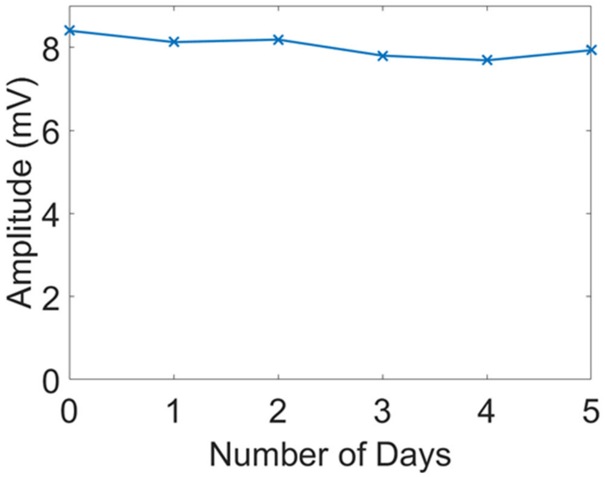

Supplementary Fig. 9. Cell Rover response over time. Amplitude of signal detected from a Cell Rover of dimension 500 µm x 200 µm x 28 µm incubated in PBS for a period of 5 days. The resonator is measured in air after incubation in PBS for the specified number of days.

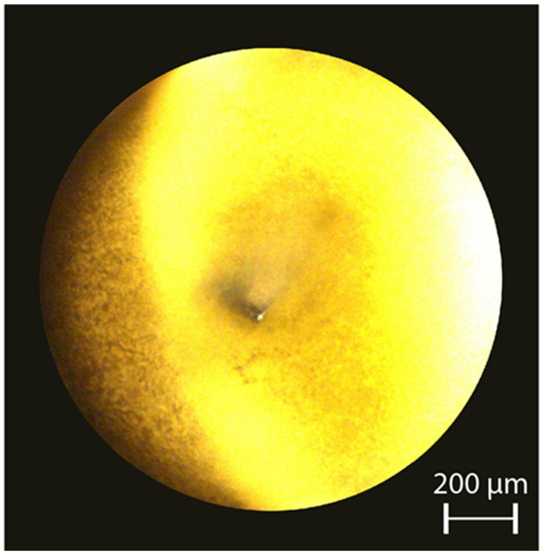

Supplementary Fig. 10. Injection of Cell Rovers under high magnetic field gradient. Optical image showing the region diametrically opposite the site of injection shortly after injection using a high magnetic field gradient. It can be seen that the antenna penetrates the cell membrane on the opposite side.

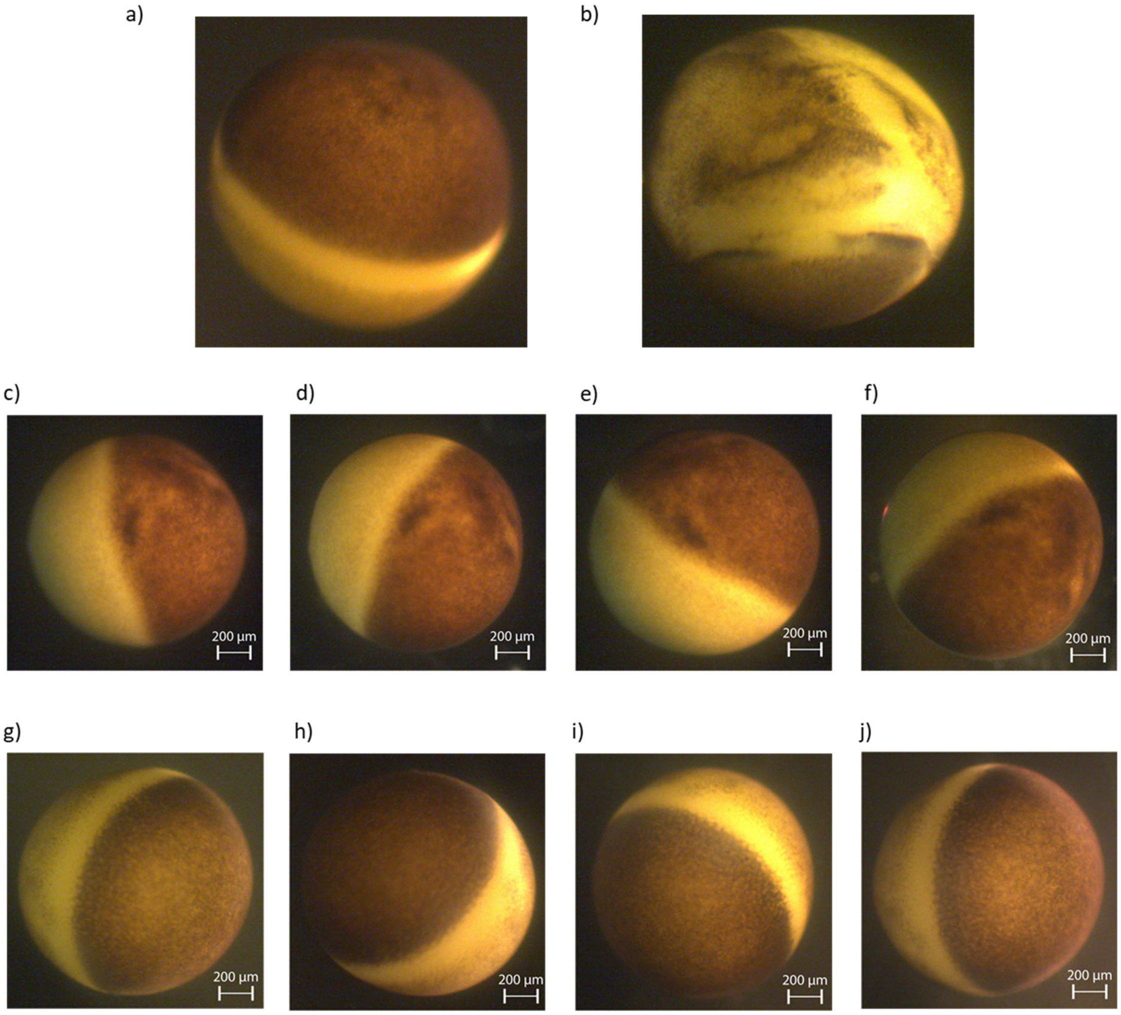

Supplementary Fig. 11. Viability of Xenopus oocytes following injection of Cell Rovers. Representative optical images showing the morphological features of **(a)** live un-injected oocytes and **(b)** dead oocytes. Optical images showing injected cell at t = **(c)**1 hr, **(d)** 2 hr, **(e)** 4 hr, **(f)** 8 hr **(g)** 12 hr, **(h)** 24 hr, **(i)** 48 hr, **(j)** 84 hr after injection respectively. The distinct brown and pale yellow hemispheres separated by the narrow unpigmented equatorial band shows that the cells are viable for long periods after injection.

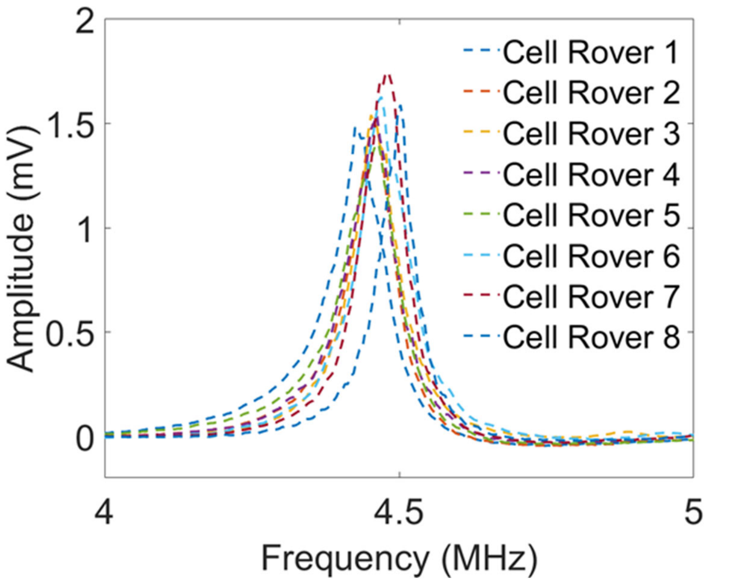

Supplementary Fig. 12. Repeatability of Cell Rover response. Measured voltage vs frequency response from 8 different Cell Rovers of sizes 500 µm x 200 µm x 28 µm injected in different Xenopus oocytes.

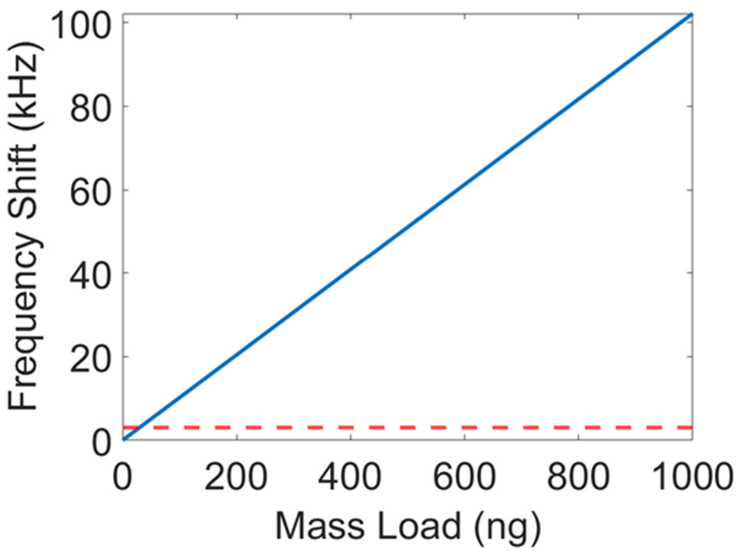

Supplementary Fig. 13. Wireless sensing using magnetostrictive resonators. Estimated shift in resonance frequency of a 500 µm x 200 µm x 28 µm resonator as a function of mass loading. The dotted line shows the minimum shift in resonance frequency which can be detected using the proposed setup.

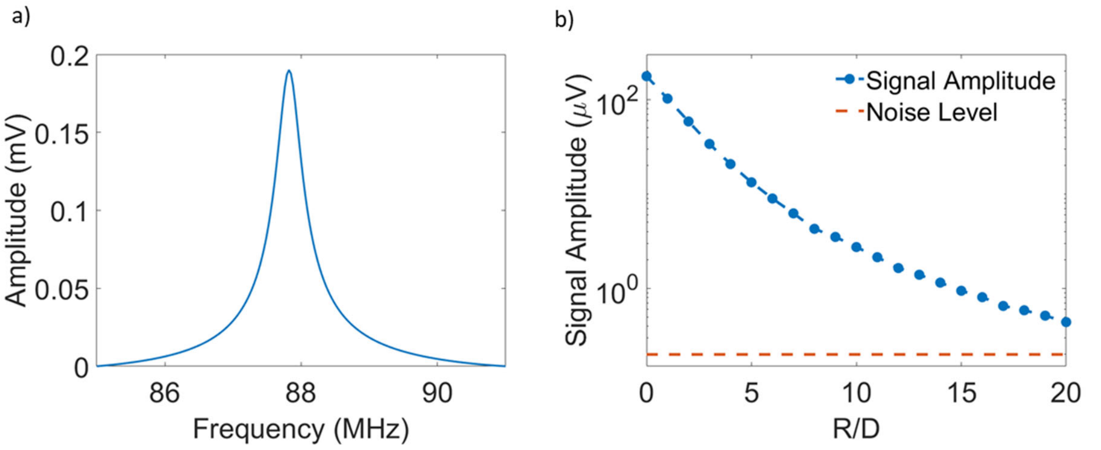

Supplementary Fig. 14. Miniaturization of Cell Rovers. (a) FEA simulated wirelessly detected voltage from a Cell Rover of dimension 25 µm x 25 µm x 5 µm in air as a function of frequency of excitation magnetic field (3 Oe). **(b)** Wirelessly detected signal amplitude from the same device as a function of *R/D*. The noise level (200 nV) is also shown. For both (a) and (b) Rx coil consists of two solenoids of 100 µm diameter and length, and 60 turns.

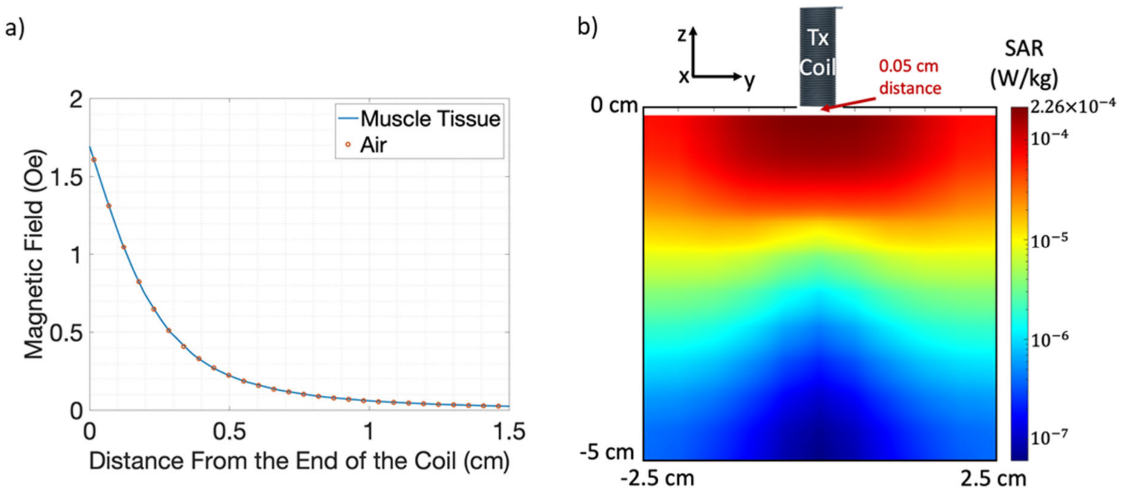

Supplementary Fig. 15. In-vivo operation of Cell Rovers. (a) CST simulation of magnetic field in air vs muscle tissue as a function distance from the end of the Tx coil. The tissue is located 0.5 mm away from the edge of coil. **(b)** CST simulation of the Specific Absorption Rate (SAR) in muscle tissue located 0.5 mm away from the edge of Tx coil. A Tx coil of 6 mm diameter, 17 mm length and 40 turns is used in both **(a)** and **(b)**.

Supplementary Table 1. Material properties of Metglas 2826 MB.

Material property	Value
Density (kg/m^3)	7900
Young's Modulus (GPa)	152.0
Magnetostrictivity (m/A)	1.19 x 10^{-8}
Poisson's Ratio	0.22
Relative Permeability (at 4.5 MHz)	300.0
Conductivity (S/m)	7.25 x 10^{5}

Supplementary Table 2. Circuit parameter values obtained for a Cell Rover of dimensions 500 μm x 200 μm x 28 μm in air, water and cell cytoplasm.

Circuit Parameter	Value (Air)	Value (Water)	Value (Cytoplasm)
L_m	$4.08\ nH$	$4.22\ nH$	$4.23\ nH$
C_m	$302.09\ nF$	$302.0\ nF$	$302.0\ nF$
R_m	$58.34\ \Omega$	$9.50\ \Omega$	$9.61\ \Omega$
k^2	1.12 %	1.12%	1.12%
L_r	5.03 nH	5.23 nH	5.23 nH

Supplementary Table 3. Calculated total coupling coefficient (k_t), quality factor (Q) and wireless power transfer efficiency (η_{opt}) for vibration in air, water and cell cytoplasm.

Parameter	Value (Air)	Value (Water)	Value (Cytoplasm)
Total Coupling Coefficient (k_t)	0.0066	0.0066	0.0066
Quality Factor(Q)	497.0	80.0	81.0
Power Transfer Efficiency(η_{opt})	3.67 %	0.62 %	0.63 %

Supplementary Table 4. Mass loading and viscous damping factor obtained from the analytical model for resonator vibration in water and cell cytoplasm.

Parameter	Value (Water)	Value (Cytoplasm)
Mass loading (m_w)($\frac{kg}{m^2}$)	0.0081	0.0080
Viscous Damping Factor(c_{visc})($\frac{kg}{m^2 s}$)	7.3×10^4	7.2×10^4

Supplementary References

1. Meeks, S. W. & Hill, J. C. Piezomagnetic and elastic properties of metallic glass alloys $Fe_{67}CO_{18}B_{14}Si_1$ and $Fe_{81}B_{13.5}Si_{3.5}C_2$. *Journal of Applied Physics* **54**, 6584–6593 (1998).

2. Zhuang, X., Leung, C. M., Li, J. & Viehland, D. Evaluation of magnetomechanical conversion efficiencies in magnetoelectric gyrators. *AIP Advances* **8**, 056607 (2017).

3. Choi, Y. S., Yoo, J. W. & Kauh, S. K. Modeling of magnetoelastic resonator using h-parameter analysis. *Journal of Mechanical Science and Technology* **30**, 749–761 (2016).

4. Yakovlev, A., Kim, S. & Poon, A. Implantable biomedical devices: Wireless powering and communication. *IEEE Communications Magazine* **50**, 152–159 (2012).

5. Yao, Z., Wang, Y. E., Keller, S. & Carman, G. P. Bulk Acoustic Wave-Mediated Multiferroic Antennas: Architecture and Performance Bound. *IEEE Transactions on Antennas and Propagation* **63**, 3335–3344 (2015).

6. Proakis, J. G. & Salehi, Masoud. Communication systems engineering. *Pearson* (2002).

7. Agcal, A., Ozcira, S. & Bekiroglu, N. Wireless Power Transfer by Using Magnetically Coupled Resonators. *Wireless Power Transfer - Fundamentals and Technologies* (2016).

8. Stoyanov, P. G. & Grimes, C. A. A remote query magnetostrictive viscosity sensor. *Sens Actuators A Phys* **80**, 8–14 (2000).

9. Green, S. R. & Gianchandani, Y. B. Wireless magnetoelastic monitoring of biliary stents. *Journal of Microelectromechanical Systems* **18**, 64–78 (2009).

10. Liu, Y. *et al.* Injectable dopamine-modified poly(ethylene glycol) nanocomposite hydrogel with enhanced adhesive property and bioactivity. *ACS Applied Materials and Interfaces* **6**, 16982–16992 (2014).

11. Cense, A. W. *et al.* Mechanical properties and failure of Streptococcus mutans biofilms, studied using a microindentation device. *J Microbiol Methods* **67**, 463–472 (2006).

12. Pasquier, D., Dupré, A. & Jessus, C. Unfertilized Xenopus eggs die by Bad-dependent apoptosis under the control of Cdk1 and JNK. *PLoS One* **6**, (2011).

13. Marchand, G. *et al.* Effects of Ferrocenyl 4-(Imino)-1,4-Dihydro-quinolines on Xenopus laevis Prophase I - Arrested Oocytes: Survival and Hormonal-Induced M-Phase Entry. *International Journal of Molecular Sciences* **21**, (2020).

14. Tang, C. Y., Chen, Y. W., Jow, G. M., Chou, C. J. & Jeng, C. J. Beauvericin activates Ca2+-activated Cl- currents and induces cell deaths in Xenopus oocytes via influx of extracellular Ca2+. *Chem Res Toxicol* **18**, 825–833 (2005).

15. Newman, K., Aguero, T. & King, M. lou. Isolation of Xenopus Oocytes. *Cold Spring Harbor Protocols* **2018**, 86–91 (2018).

16. Siegfried F, G. *et al.* Fully automated microinjection system for Xenopus laevis oocytes with integrated sorting and collection. *J Lab Autom* **16**, 186–196 (2011).
