# Supplementary material for: Cell Rover—a miniaturized magnetostrictive antenna for wireless operation inside living cells
Source: Nat Commun. 2022 Sep 22;13:5210. doi: 10.1038/s41467-022-32862-4 (PMC9499948; doi:10.1038/s41467-022-32862-4)
Supplement: Supplementary file 2 — Description of Additional Supplementary Files [file 41467_2022_32862_MOESM2_ESM.pdf]

Description of Additional Supplementary Files

File Name: Supplementary Movie 1
Description: Time domain response of resonator.

File Name: Supplementary Movie 2
Description: Intracellular injection of Cell Rovers.
